# Cardiac remodelling in type 2 diabetes: Pathophysiological mechanisms and opportunities for multiscale computational modelling and simulation

**DOI:** 10.1113/JP287140

**Published:** 2026-01-02

**Authors:** Ambre Bertrand, Jakub Tomek, Blanca Rodriguez

**Affiliations:** ^1^ Department of Computer Science University of Oxford Oxford UK; ^2^ Department of Physiology, Anatomy & Genetics University of Oxford Oxford UK

**Keywords:** cardiac arrhythmias, cardiac electrophysiology, cardiac mechanics, computational modelling and simulation, diabetic myocardial disorder, *in silico* trials, precision medicine, type 2 diabetes

## Abstract

Type 2 diabetes is a highly prevalent metabolic disease that significantly impacts the heart and contributes to an increased risk of cardiac complications, notably heart failure with preserved ejection fraction and cardiac arrhythmias, which can cause sudden cardiac death. In type 2 diabetes chronic hyperglycaemia and insulin resistance lead to subcellular changes, including dysregulation of calcium/calmodulin‐dependent protein kinase II (CaMKII), intracellular sodium and calcium handling and potassium currents, all of which impair cardiac contractility and repolarisation. Type 2 diabetes induces diffuse myocardial fibrosis and anatomical remodelling, which contribute to diastolic and systolic dysfunction, and the formation of a pro‐arrhythmic substrate. Impaired connexin 43‐mediated conduction and cardiac autonomic neuropathy further promote cardiac electrophysiological and mechanical dysfunction. Clinical studies using the ECG and cardiac imaging modalities have been successful in detecting some of these changes; however our mechanistic understanding of type 2 diabetes‐driven cardiac disorders remains limited. Recent advances in multiscale computational modelling and simulation of human cardiac electrophysiology and mechanics provide new opportunities to study diabetes‐induced cardiac remodelling *in silico* by unravelling disease mechanisms across different scales and assisting in the development of novel therapies. Here we review key pathophysiological mechanisms of electrophysiological, structural and nervous cardiac remodelling in type 2 diabetes; their clinical implications; and the cardiac effects of common glucose‐lowering pharmacological agents commonly taken by diabetes patients. We discuss the potential of human‐based computational cardiac modelling and simulation in this context to deepen our mechanistic understanding, and guide more precise prevention and treatment of diabetes‐driven cardiac arrhythmias and diastolic dysfunction.

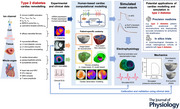

## Graphical abstract

**Abstract figure legend** Overview of multiscale cardiac remodelling in type 2 diabetes and how to model and simulate these changes using a human‐based, multiscale computational framework. Cardiac remodelling in type 2 diabetes occurs at ionic channel, protein, cellular, tissue and whole‐organ level, affecting the electrophysiological function, mechanical function and structure of the diabetic heart. Experimental and clinical data capture some of these changes, as illustrated. These data can be integrated into biophysically detailed patient‐specific computational models of the heart to enable the simulation of pathophysiological mechanisms across different scales, creating a testbed for hypothesis generation and testing, and *in silico* pharmacological therapy evaluation. This figure was created using icons from Biorender, with visuals adapted in part from Chen et al. (2022), Coleman et al. (2024), Doste et al. (2019). APD, action potential duration; CaMKII, calcium/calmodulin‐dependent protein kinase II; HR, heart rate; HRV, heart rate variability; LV, left ventricular; MIBG, iodine meta‐iodobenzylguanidine; MRI, magnetic resonance imaging; SPECT, single‐photon emission computed tomography.

## Introduction and clinical implications

Type 2 diabetes (T2DM) is a complex metabolic disease that affects over half a billion adults globally (Sun et al., 2022). Its development is influenced by diet, body weight and age; its prevalence increasing steadily due to obesity and ageing populations. T2DM is characterised by chronically high levels of blood glucose linked to impaired insulin secretion and insulin resistance, which cause abnormal glucose absorption from the blood into different organs. Insulin being the only hormone that lowers blood glucose concentration these combined factors contribute to chronic hyperglycaemia, the central trigger responsible for a cascade of adverse mechanisms affecting the heart.

Individuals with T2DM have a two‐ to four‐fold increased risk of cardiovascular disease, the leading global cause of death (Marx et al., 2023). More specifically T2DM is associated with a higher risk of cardiac arrhythmias, including atrial fibrillation (Aksnes et al., 2008; Benjamin et al., 1994; Huxley et al., 2012; Rawshani et al., 2023) and ventricular arrhythmias (Chakraborty et al., 2024; Jungen et al., 2019; Rawshani et al., 2023; Scheen, 2022), both of which link to sudden cardiac death (SCD). Notably T2DM patients have a two‐ to fourfold increased risk of SCD (Bergner & Goldberger, 2010; Ro et al., 2016). T2DM is also a major risk factor for heart failure with preserved ejection fraction (HFpEF), a heterogenous and highly comorbid syndrome that affects over 60 million people, and is extremely challenging to treat (Anker et al., 2023; Savarese et al., 2023). Arrhythmic risk is further increased in patients with concomitant HFpEF and T2DM, which make up 20%–45% of HFpEF cases (MacDonald et al., 2008; Mentz et al., 2014; Ruwald et al., 2013). Silent myocardial infarction and ischaemia are also more common in T2DM, further increasing the risk of heart failure, coronary artery disease and death (Arenja et al., 2013; Singleton et al., 2020; Soejima et al., 2019).

T2DM is associated with ECG abnormalities reflecting underlying electrophysiological remodelling. QT segment and T‐wave irregularities, notably QT prolongation and T‐wave flattening, are common in T2DM even without overt heart failure or ischaemia (Bertrand, Lewis, et al., 2024; Kuzu, 2018; Mould et al., 2021; Pappachan et al., 2008; Soflaei Saffar et al., 2024; Stern & Sclarowsky, 2009; Tokatli et al., 2016). T‐wave alternans and increased QT‐interval dispersion are also frequent in T2DM and are linked to a higher incidence of arrhythmias and higher mortality (Molon et al., 2007; Naas et al., 1998; Perkiömäki et al., 2015; Sawicki et al., 1998; Veglio et al., 2002). Clinical imaging modalities such as cardiac magnetic resonance (CMR) and echocardiography can identify subclinical changes at organ level in the diabetic myocardium, including a thicker left ventricular wall, smaller chamber volumes, diastolic dysfunction and myocardial fibrosis (Bertrand, Lewis, et al., 2024; Devereux et al., 2000; Jia et al., 2016; Levelt et al., 2016).

However, the mechanisms underlying SCD and clinically observed changes in cardiac function in T2DM are complex and remain poorly understood, hindering the development of novel targeted therapies. These therapies would ideally go beyond current approaches focused on systemic glucose control or symptomatic HFpEF management, which primarily aim to alleviate haemodynamic burden rather than address arrhythmogenic substrates. A deeper mechanistic understanding of how T2DM alters myocardial electrophysiology and structure could enable new therapeutic strategies directed at preventing malignant arrhythmias and SCD, complementing existing pharmacological and device‐based interventions for HFpEF. A general complication in designing new, mechanism‐informed anti‐arrhythmic strategies is the heterogeneity of animal models (including age, animal species and strain (Hayashi et al., 2006), mode of T2DM induction, duration of disease, concomitant conditions) coupled with the fact that experimental models of T2DM are predominantly rat and mice models, which differ significantly from human. Recent technological advances now allow to augment experimental and clinical data through computational methods, including artificial intelligence‐enabled multiscale modelling and simulation of human pathophysiology. These methods have significantly progressed in cardiac electrophysiology and mechanics, enabling human‐based computer cardiac simulations from subcellular to whole‐organ behaviour, including the manifestation of abnormalities in the ECG and pressure‐volume loops (Corral‐Acero et al., 2020; Lee et al., 2021; Salvador et al., 2024; Tomek et al., 2019; Zhou et al., 2024). These novel approaches have been widely and successfully applied to different cardiac diseases such as atrial fibrillation (Dasí et al., 2022; Heijman et al., 2021), myocardial ischaemia and infarction (Arevalo et al., 2016; Martinez‐Navarro et al., 2019; Niederer et al., 2019; Zhou et al., 2024), hypertrophic cardiomyopathy (Coleman et al., 2024; Lyon et al., 2018), as well as therapeutic approaches such as pharmacological treatment and ablation (Boyle et al., 2019; Dasí et al., 2024) of atrial fibrillation, stem cell injection (Riebel et al., 2024) and cardiac resynchronisation therapy (Strocchi, Samways, et al., 2025). The potential of those methods to model and simulate the human heart under diabetic conditions and relevant therapies, however, remains largely untapped. Such virtual human‐based investigations would also help to overcome the barrier to translation represented by the widespread use of small animal models in T2DM‐based cardiac experimental research. Finally, a major advantage of comprehensive multiscale modelling and simulation frameworks is their capability to capture multiple relevant outputs. Although searching for anti‐arrhythmic treatments one may concurrently keep track of predicted effects of candidate drugs on muscle contraction and relaxation properties, making sure that mechanical function is not affected adversely. Likewise, strategies to improve mechanical function may be monitored for the predicted arrhythmic risk, promoting broad‐scale safety and efficacy.

This review summarises key mechanisms of cardiac electrophysiological, structural and nervous remodelling driven by T2DM across different biophysical scales, highlighting key experimental findings and relevant clinical evidence. We also discuss the cardiac effects of common pharmacological treatments that target primarily glucose control in T2DM and their implications on arrhythmic risk and cardiac function. Finally, we present novel human‐based computational modelling and simulation approaches that could help unravel disease mechanisms and enable *in silico* trials in diabetes‐driven cardiac disorders.

## Structural remodelling at tissue and whole‐organ level

The structural and functional effects of diabetes‐induced cardiac remodelling at whole‐organ level, which closely resemble heart failure, have been formally referred to as diabetic cardiomyopathy since the 1970s (Rubler et al., 1972). This condition was defined as the occurrence of myocardial structural and/or functional abnormalities associated with T2DM in the absence of coronary heart disease, hypertension and/or obesity. Given that the majority of T2DM patients nowadays have one or more of these contributing comorbidities this definition was recently relaxed. The condition is now referred to as diabetic myocardial disorder and is considered a T2DM‐specific pre‐heart failure stage that warrants careful clinical attention (Seferović et al., 2024). In this section we discuss some of the hallmarks of diabetic myocardial disorder at tissue and whole‐organ level.

### Anatomical remodelling

Patients with T2DM tend to have a higher left ventricular mass (Devereux et al., 2000; Eguchi et al., 2008) and wall thickness (Bertrand, Lewis, et al., 2024; Devereux et al., 2000). These changes worsen as the disease progresses (Lee et al., 1997; Parsa et al., 2023). Overt left ventricular hypertrophy is frequently observed in patients with T2DM (Eguchi et al., 2008), characterised by an increased left ventricular mass to left ventricular end‐diastolic volume ratio but a normal left ventricular mass index (Levelt et al., 2016). Some proposed mechanisms for this observation include cardiac steatosis, whereby hypertrophic signalling and left ventricular concentric remodelling are enhanced in conditions of lipid accumulation; hyperactivation of the insulin signalling cascade in patients with obesity and T2DM patient and increased levels of circulating pro‐inflammatory cytokines (Cook et al., 2010; Karason et al., 2003). Concentric remodelling refers to an increase in left ventricular relative wall thickness with a reduction in chamber size. As left ventricular wall thickness increases, the left ventricular end‐diastolic and end‐systolic volumes typically decrease, reflecting a geometric adaptation rather than dilatation. This inward concentric remodelling and associated ventricular chamber changes lead to a reduced blood pool size while the external cardiac dimensions remain mostly unchanged.

Recent modelling work proposed a unified framework linking left ventricular geometry and tissue properties with pressure‐volume relations, enabling analysis of cardiac function both under physiological loading and from purely geometric data (Zhang et al., 2023). Such frameworks could help bridge structural remodelling in T2DM with biomechanical and haemodynamic function.

### Myocardial fibrosis

**Figure 1:**
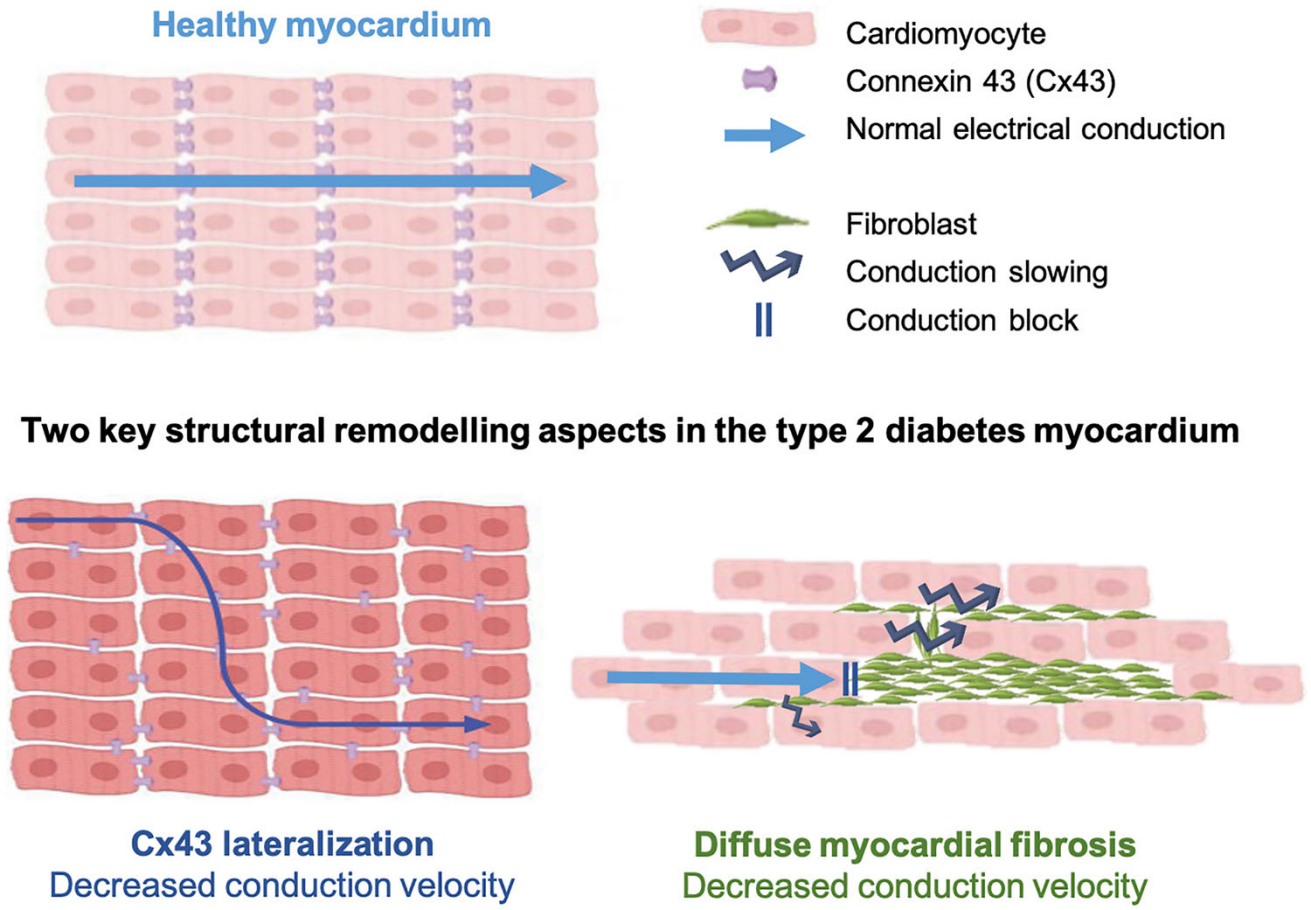
Effect of diffuse myocardial fibrosis and connexin 43 (Cx43) lateralisation on electrical conduction velocity in the heart Schematics adapted in part from Cho (2022).

At tissue level, cardiac remodelling in T2DM is mainly characterised by the presence of diffuse interstitial myocardial fibrosis. In hyperglycaemia, the increased production of reactive oxygen species and advanced glycation end products cause cross‐linkage of extracellular matrix proteins in the myocardium (Aronson, 2003; Pappachan et al., 2013). Collagen, the key structural component of the extracellular matrix, is non‐conductive, hindering electrical wave propagation (Fig. 1). Excess or highly cross‐linked collagen increases myocardial stiffness and reduces contractility, however some collagen production and cross‐linking are essential for structural integrity; too little can weaken the myocardium, as shown experimentally by Rainer et al., who demonstrated that broad transforming growth factor‐β (TGF‐β) blockade, suppressing fibroblast activation and collagen synthesis, led to increased mortality from wall rupture (Rainer et al., 2014). Additional sources of diffuse fibrosis in T2DM include the activation of cardiac fibroblasts into myofibroblasts, impaired autophagy of apoptotic or necrotic cells and elevated sympathetic nervous system activity, which enhances β_1_‐adrenergic receptor signalling (Gibb et al., 2020; Jia et al., 2016, 2018). A post‐mortem study identified diffuse fibrosis throughout the myocardium of four middle‐ to old‐age diabetic patients with a history of diabetes of 5 or more years (Rubler et al., 1972). A CMR study showed that in T2DM patients diffuse fibrosis is distributed heterogeneously in the ventricles, with more severe fibrosis present in the septum and inferolateral walls (Bojer et al., 2024). Using H(1) magnetic resonance spectroscopy, Levelt et al. showed that cardiac steatosis is increased in T2DM patients (Levelt et al., 2016). Another CMR study showed that epicardial adipose tissue, which increases with body mass index (BMI), is positively associated with interstitial myocardial fibrosis and cardiac steatosis (Ng et al., 2018). Interstitial fibrosis and epicardial adipose tissue both contribute to contractile dysfunction and an increased likelihood of arrhythmias via delayed conduction and the formation of re‐entry circuits (Ernault et al., 2021; Ten Tusscher & Panfilov, 2007).

### Diastolic and systolic dysfunction

As explained above, cardiac fibrosis causes tissue to be stiffer, impairing normal contraction and relaxation of the myocardium in T2DM. Initially, this manifests as substantial changes in diastolic function and may lead to systolic dysfunction at later, more severe stages of T2DM (Pappachan et al., 2013). Impaired myocardial relaxation can be identified at early stages by CMR by a decrease in initial and peak filling rates, or by echocardiography by abnormal wall motion and longer isovolumic relaxation times (Jia et al., 2016). At later stages, coronary microcirculation becomes further impaired, and diastolic and systolic dysfunction become more severe. Indeed, in advanced T2DM, diastolic dysfunction and other symptoms start resembling the HFpEF phenotype more closely (Tan et al., 2020). In addition to structural causes of diastolic dysfunction, abnormal Ca^2+^ handling in T2DM with higher [Ca^2+^]_i_ levels and longer [Ca^2+^]_i_ decay leads to prolonged relaxation times; these cellular‐level changes will be discussed further in a later section.

## Electrophysiological remodelling at tissue and whole‐organ level

As discussed in the previous section, diffuse interstitial myocardial fibrosis represents an important substrate for cardiac arrhythmias in T2DM. Remodelling of connexin 43 (Cx43) proteins and damage to the systemic and intrinsic cardiac nervous systems further exacerbate the arrhythmic substrate in T2DM.

### Connexin 43 and impaired conduction

Along with their role in the formation of fibrosis, myofibroblasts express gap junction connexin proteins Cx43 and Cx45, which enable them to directly modulate cardiomyocytes’ electrophysiological behaviour, including a reduction in cardiac impulse conduction (Miragoli et al., 2006). Cx43 appears to be lateralised in T2DM, i.e. redistributed along the lateral sides of cardiomyocytes rather than the cell–cell junctions, and associated with non‐functional gap junctions (Nygren et al., 2007) (Fig. 1). Furthermore, Cx43 expression levels are reduced overall in diabetes (Lin et al., 2006); this reduction is spatially heterogeneous in the ventricles (Billur et al., 2022). Analogous findings on the spatially heterogenous reduction in Cx43 have been reported in studies of related myocardial disorders such as heart failure in humans (Dupont et al., 2001) and hypertrophy in rats (Boulaksil et al., 2016). These Cx43 alterations are associated with heterogeneities in resting membrane potentials, action potential duration (APD) and refractory period, leading to spatially dispersed current conduction in the myocardium, and thus to an enhanced vulnerability to ventricular arrhythmias, even in the absence of fibrosis (Severs et al., 2008; Yan et al., 2021). These changes may explain the QT dispersion observed clinically in the ECG.

### Cardiac autonomic dysfunction

Chronic hyperglycaemia is responsible for the progression of cardiac autonomic neuropathy, another common cardiac complication of T2DM which affects the intrinsic cardiac nervous system (Di Carli et al., 1999; Goldberger et al., 2019). The intrinsic cardiac nervous system is made up of post‐stellate ganglionic, sympathetic nerve fibres and parasympathetic nerve fibres descending from the vagus nerve (Giannino et al., 2024). Neurons emerging from the intrinsic cardiac nervous system cluster in distinct areas of the heart to form various ganglionated intracardiac plexi (Armour et al., 1997; Pauza et al., 2000). In T2DM, intracardiac ganglia undergo structural and functional remodelling, while the cardiac nerves connected to the parasympathetic fibres at the atrioventricular and sinoatrial nodes are damaged progressively from the apex of the ventricles to the base (Evans & Li, 2024; Vinik et al., 2011).

In the early stages of T2DM‐induced cardiac autonomic neuropathy, parasympathetic function decreases relative to sympathetic function which increases, causing heart rate variability to decrease (Draman et al., 2013; Vinik et al., 2013). Decreased heart rate variability has long been known to be associated with a heightened risk of adverse cardiac events (Tsuji et al., 1996). Heightened sympathetic activity stimulates the renin‐angiotensin‐aldosterone system and increases heart rate, stroke volume and vascular resistance, leading to high blood pressure and ventricular dysfunction (Mustonen et al., 1992; Perin et al., 2001). At later stages, resting tachycardia is common, as well as both severely impaired heart rate variability and blunted cardiac output response following exercise, indicating almost complete cardiac denervation (Vinik et al., 2013). Cardiac denervation in T2DM is likely to blunt patients’ perception of myocardial pain, explaining the higher risk of silent myocardial ischaemia and further subclinical cardiac decline (Draman et al., 2013). Cardiac denervation may also explain the higher rates of atrioventricular block and bundle‐branch block in T2DM (Eriksson et al., 1998; Haxha et al., 2023; Movahed et al., 2023). This could be explained by the damaged atrioventricular and sinoatrial nodes, which may have a downstream impact on electrical signal propagation through the bundle of His and downstream Purkinje fibres responsible for the control and rapid distribution of electrical activity through the ventricles.

Cardiac sympathetic denervation has been shown to correlate with diffuse fibrosis and to modulate arrhythmic risk in non‐ischaemic cardiomyopathies, including diabetic cardiomyopathy (Chen et al., 2022). Even in the absence of overt nerve fibre degeneration, sympathetic dysfunction is associated with an increased susceptibility to ventricular arrhythmias in mice with T2DM (Jungen et al., 2019). The imbalance of sympathetic and parasympathetic activity causes an increase in dispersion of electrical activation and repolarisation in the ventricles from the endocardium to epicardium, creating a more pro‐arrhythmic setting (Goldberger et al., 2019) through conduction block and/or re‐entry, resulting from this slowed or heterogenous impulse propagation. This vulnerability is further amplified by the tissue‐level heterogeneity established by fibrosis through disrupted intracellular electrical coupling and increased conduction dispersion. So, while both mechanisms coexist, nervous conduction system damage can act as a functional pro‐arrhythmic trigger, with fibrosis exacerbating the structural vulnerability of the substrate.

Clinically, characteristic autonomic effects may be reflected in the ECG, notably with the QT‐RR slope and QTc interval prolongation (Goldberger et al., 2019; Pappachan et al., 2008), increase/decrease in heart rate relating to increase/decrease in sympathetic/parasympathetic activity, respectively, as explained above, and reduced heart rate variability reflecting poorer autonomic modulation and increased risk of cardiac events (Draman et al., 2013; Tsuji et al., 1996; Vinik et al., 2013). Cardiac denervation and the sympathetic nervous system can be assessed using single‐photon emission computed tomography imaging with an iodine‐123 meta‐iodobenzylguanidine radioactive tracer (Chen et al., 2022).

## Subcellular electrophysiological remodelling and calcium handling

While electrophysiological disturbances in T2DM can be observed clinically in the ECG, notably QT interval prolongation and T wave changes, experimental studies remain crucial to unravel the underlying mechanisms responsible for those changes at ionic level and how they may trigger cardiac arrhythmias.

Ashrafi et al. collected ventricular tissue mRNA data from patients with T2DM and translated these results to relative ionic current changes (Ashrafi et al., 2017). They found an increase in L‐type calcium current (I_CaL_), sodium–calcium exchanger current (I_NaCa_), sodium current (I_Na_), late sodium current (I_NaL_); a decrease in slow and rapid delayed rectifier potassium currents (I_Ks_, I_Kr_), transient outward potassium current (I_to_); and a decrease in SERCA pump flux (J_up_) and ryanodine receptor activity (J_rel_) (see Tables 1 and 2). Multiple animal studies on ionic current changes in T2DM have been conducted; however results are heterogenous (Gallego et al., 2021).

**Table 1:** Experimental data of ionic channels remodelling observed in cardiac ventricular myocytes of humans and/or animals with type 2 diabetes

Ionic current	Reference	Change from baseline (approx.)	HbA1c (mmol mol^−1^, mean ± SD)	Blood glucose (mg/dl, mean ± SD)	Experimental model and mean age	Experimental protocol and conditions
I_Na_	(Ashrafi et al., 2017) (Table 6)	+21.08%	57.9 ± 14.57	‐	Human (*n* = 7), 74.9 years old	Nav1.5 mRNA expression by quantitative PCR
I_to_	(Ashrafi et al., 2017) (Table 6)	+26.04%	57.9 ± 14.57	‐	Human (*n* = 7), 74.9 years old	Kv 1.4, 4.2 and 4.3 mRNA expression by quantitative PCR
I_CaL_	(Ashrafi et al., 2017) (Table 6)	+14.18%	57.9 ± 14.57	‐	Human (*n* = 7), 74.9 years old	Cav1.2 and 1.3 mRNA expression by quantitative PCR
I_CaL_	(Pereira et al., 2006) (Fig. 2)	−4pA/pF (−31.2%)	‐	‐	Db/db mice (*n* = 20), 15 weeks old	Patch‐clamp, H*P* = 100 mV, voltage steps to 20 mV, >180 sweeps per experiment. Recorded at room temperature (21–23°C)
I_CaL_	(Pereira et al., 2006) (Fig. 3)	−38%	‐	‐	Db/db mice (*n* = 5), 15 weeks old	Expression of pore‐forming 1Cα subunit of LTCC by western blotting
I_Kr_	(Ashrafi et al., 2017) (Table 6)	−65.24%	57.9 ± 14.57	‐	Human (*n* = 7), 74.9 years old	ERG mRNA expression by quantitative PCR
I_Ks_	(Ashrafi et al., 2017) (Table 6)	−5.38%	57.9 ± 14.57	‐	Human (*n* = 7), 74.9 years old	KvLQT1 mRNA expression by quantitative PCR
I_NaL_	(Z. Lu et al., 2013) (Fig. 2)	+0.1pA/pF (+100%)	‐	>500	Db/db mice (*n* = 7), 2.5 months old	Current elicited by 750 ms depolarising voltage steps ranging from H*P* = −80 to 0 mV at 10 mV increments. Recorded at room temperature

*Note*: Units of change from baseline are indicated in each row depending on the type of data, percentage change for protein expression data and pA/pF for ionic currents.

Abbreviations: HP, holding potential; LTCC, L‐type calcium channel; STZ, streptozotocin.

**Table 2:** Experimental data of ionic current or ionic flux remodelling associated with a Ca^2+^ handling protein (RyR, SERCA2, NCX) observed in cardiac ventricular myocytes of humans and/or animals with type 2 diabetes

Ionic current/flux	Reference	Change from baseline (approx.)	HbA1c (mmol mol^−1^, mean ± SD)	Blood glucose (mg/dl, mean ± SD)	Experimental model and mean age	Experimental protocol and conditions	
I_NaCa_ (NCX)	(Ashrafi et al., 2017) (Table 6)	+243.83%	57.9 ± 14.57	‐	Human (*n* = 7), 74.9 years old	NCX1 mRNA expression by quantitative PCR	
I_NaCa_ (NCX)	(Hattori et al., 2000)	−5%	‐	605 ± 9	STZ rat (*n* = 26), 12–14 weeks, 8 weeks old at STZ injection	Whole‐cell voltage clamp, ramp pulses from H*P* = −60 mV to +50 mV, then −100 mV to −60 mV, at steady speed of 0.67 V s−1. Recorded at 36±1°C	
I_NaCa_ (NCX)	(Stolen et al., 2009)	+59%	‐	362 ± 16	Db/db mice (*n* = 20 exercise, 20 sedentary), 7 weeks old	Fura‐2/AM–loaded myocytes; caffeine‐induced SR Ca^2^⁺ release (SERCA2a inhibited); Ca^2^⁺ decay kinetics used to quantify NCX‐mediated extrusion
J_rel_ (RyR)	(Ashrafi et al., 2017) (Table 6)	−10.07%	57.9 ± 14.57	‐	Human (*n* = 7), 74.9 years old	RYR2 mRNA expression by quantitative PCR	
J_rel_ (RyR)	(Teshima et al., 2000) (Fig. 2)	−38%	‐	773 ± 17	STZ rat (*n* = 4), ‘mature’, data collected 12 weeks post‐STZ injection	RYR mRNA expression by Northern blotting	
J_rel_ (RyR)	(Netticadan et al., 2001) (Fig. 1)	−45%	‐	322 ± 24	STZ rat (*n* = 4), undisclosed age, data collected 6 weeks post‐STZ injection	RYR mRNA expression by western blotting	
J_up_ (SERCA2)	(Pereira et al., 2006)	−1.3 Ca^2+^ sparks/s/100 µm (−34%)	‐	‐	Db/db mice (*n* = 14), 15 weeks old	Line‐scan recording in saponin‐permeabilised myocytes. Recorded at room temperature (21–23°C)	
J_up_ (SERCA2)	(Belke et al., 2004) (Fig. 5)	−13%	‐	489 ± 44	Db/db mice (*n* = 9), 12 weeks old	SERCA 2a expression by western blotting	
J_up_ (SERCA2)	(Zhang et al., 2008) (Fig. 4)	−50%	‐	553.2 ± 16.2	STZ Wistar rat (*n* = 8), ‘adult’	SERCA 2a expression by western blotting	

*Note*: Units of change from baseline are indicated in each row depending on the type of data.

Abbreviations: HP, holding potential; RyR, ryanodine receptors; STZ, streptozotocin.

**Figure 2:**
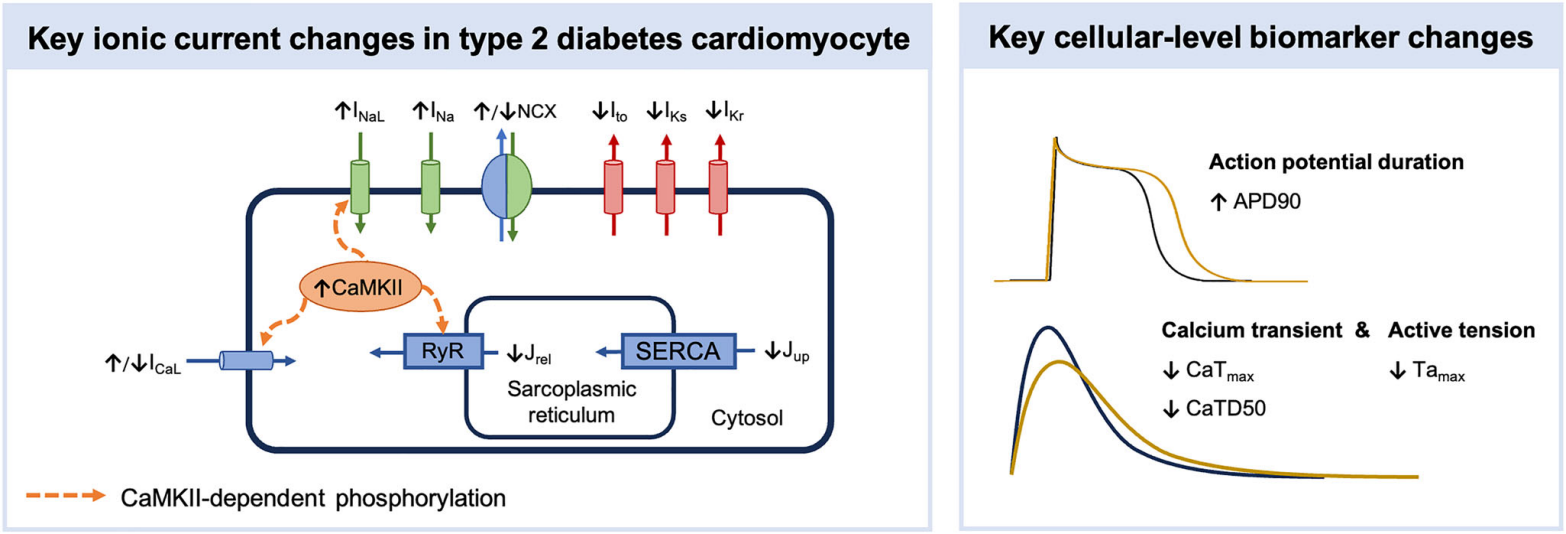
Summary of subcellular‐level changes in a type 2 diabetes ventricular cardiomyocyte Increase and decrease in ionic currents and calmodulin‐dependent protein kinase II (CaMKII) are indicated by up and down arrows, respectively. Factors most likely to drive the APD prolongation are predominantly the increase in I_NaL_, I_NCX_ and I_CaL_ when present, as well as the reduction in I_Kr_ and I_Ks_. The reduced calcium transient amplitude is multifactorial, with likely key factors being the increased NCX activity, reduced SERCA pumps and increased SR leak, although these are partly offset by increased I_NaL_ and longer calcium influx via I_CaL_, a consequence of the prolonged APD. APD, action potential duration; CaT, intracellular calcium transient; D50, duration at half‐maximum amplitude; SR, sarcoplasmic reticulum; Ta, active tension.

In this section, we review important mechanisms underlying subcellular electrophysiological changes in T2DM and their implications on arrhythmic risk and cellular contractility. Key changes in ionic current and calcium handling protein remodelling; calmodulin‐dependent protein kinase II (CaMKII) activity; and action potential, calcium transient and contractility biomarkers recorded in experimental studies of T2DM are summarised in Tables 1, 2, 3, 4, respectively. Fig. 2 provides an illustrative overview of these changes.

**Table 3:** Experimental data of CaMKII activity observed in cardiac ventricular myocytes of humans and/or animals with type 2 diabetes

CaMKII activity	Reference	Change from baseline (approx.)	Experimental model	Experimental protocol and conditions
Relative CaMKII activity	(Erickson et al., 2013)	+110%	HIP rat (*n* = 3)	See Despa et al. (2012)
Autonomous CaMKII activation	(Erickson et al., 2013)	+40%	HIP rat (*n* = 3), glucose 240 mg/dl^−1^	
		+70%	HIP rat (*n* = 3), glucose 500 mg/dl^−1^	
				Ratio of fluorescence of CFP to YFP
CaMKII activation state in cardiac myocytes following increased [Ca^2+^]_i_	(Erickson et al., 2013)	+10%	Human (*n* = 6), glucose 100 mg/dl^−1^	0.5 Hz pacing
		+20%	Human (*n* = 6), glucose 200 mg/dl^−1^	0.5 Hz pacing
		+25%	Human (*n* = 9), glucose 100 mg/dl^−1^	Isoprenaline (100 nM for 20 min)
		+30%	Human (*n* = 9), glucose 200 mg/dl^−1^	Isoprenaline (100 nM for 20 min)
				Ratio of fluorescence of CFP to YFP
CaMKII phosphorylation	(Daniels et al., 2018)	+82%	ZDF rat (*n* = 8)	CaMKII band intensities normalised to GAPDH, ratio of phosphorylated CaMKII relative to total CaMKII measured

*Note*: Units of change from baseline are indicated in each row depending on the type of data.

Abbreviations: CFP, cyan fluorescent protein; HP, holding potential; HIP, human islet amyloid polypeptide; STZ, streptozotocin; YFP, yellow fluorescent protein; ZDF, Zucker diabetic fatty rat.

**Table 4:** Action potential duration, calcium transient and active tension biomarkers observed in cardiac ventricular myocytes of humans and/or animals with type 2 diabetes

Biomarker	Reference	Change from baseline (approx.)	Experimental model	Experimental protocol and conditions
APD90	(Lu et al., 2013)	+130 ms	Db/db mice (*n* = 20), glucose >500 mg/dl^−1^	‐
		±20ms	Db/db mice (*n* = 10), glucose >500 mg/dl^−1^	PI3K messenger, PIP3
		±20ms	Db/db mice (*n* = 7), glucose >500 mg/dl^−1^	I_NaL_ blocker mexiletine (4 mg/ml for 2 h)
				Pulse amplitude = 120 pA, *t* = 10 ms, cycle length = 1 s. Recorded at room temperature
APD90	(Hegyi et al., 2021)	+15 ms	Mouse ventricle, glucose 30 mM 6 min	‐
		±0 ms	Rabbit ventricle, glucose 30 mM 6 min	‐
		−60 ms	Rabbit ventricle, glucose 30 mM 6 min	I_NaL_ inhibitor GS‐967 (1 µM)
		±0 ms	Mouse ventricle, glucose 30 mM 6 min	CaMKII inhibitor AIP (1 µM)
				1 Hz steady‐state pacing
APD90	(Zhang et al., 2008)	−59 ms (−30%)	STZ Wistar rat (*n* = 7)	0.1–7 Hz stimulation frequencies, 37°C
APD90	(Zhang et al., 2008)	−36ms (−37%)	STZ Wistar rat (*n* = 7)	0.1–7 Hz stimulation frequencies, 37°C
CaT_max_	(Pereira et al., 2006)	−0.7 F/F0	Db/db mice (77 cells from *n* = 4 hearts)	Fluorescence recorded with MetaZeiss LSM510, objective water immersion 60, numerical aperture 1.2, in line‐scan mode (1.5 ms/line)
CaT_max_	(Belke et al., 2004)	−50%	Db/db mice (*n* = 9)	Solomere Technologies system configured for dual emission, Nikon Diaphot epifluorescence microscope, recorded at 100 Hz, room temperature
CaT, D50	(Pereira et al., 2006)	+72.8 ms	Db/db mice (*n* = 4)	Fluorescence recorded with MetaZeiss LSM510, objective water immersion 60, numerical aperture 1.2, in line‐scan mode (1.5 ms/line)
CaT, D50	(Belke et al., 2004)	+33%	Db/db mice (*n* = 9)	Solomere Technologies system configured for dual emission, Nikon Diaphot epifluorescence microscope, recorded at 100 Hz, room temperature
Ta_max_	(Zhang et al., 2008)	−7 mN/mm^2^ (−41%)	STZ Wistar rat (*n* = 8)	1.5 mmol/l [Ca^2+^]_o_, 37°C and 5 Hz
Ta, TTP	(Zhang et al., 2008)	+10 ms (+15%)	STZ Wistar rat (*n* = 9)	1.5 mmol/l [Ca^2+^]_o_, 37°C and 5 Hz
Ta, RT50	(Zhang et al., 2008)	+10 ms (+31%)	STZ Wistar rat (*n* = 9)	1.5 mmol/l [Ca^2+^]_o_, 37°C and 5 Hz
Ta, RT90	(Zhang et al., 2008)	+20 ms (+36%)	STZ Wistar rat (*n* = 9)	1.5 mmol/l [Ca^2+^]_o_, 37°C and 5 Hz

Note: 1 mN/mm^2^ = 1 kPa.

Abbreviations: APD, action potential duration; CaT, intracellular calcium [Ca^2+^]_i_ transient; D50, duration at half‐maximum amplitude; RT50, relaxation time as peak time to 50% decay; RT90, relaxation time as peak time to 90% decay; STZ, streptozotocin; Ta, active tension; TTP, time to peak.

### CaMKII‐mediated sodium and calcium dysregulation

Hyperglycaemia activates the hexosamine biosynthetic pathway, further leading to post‐translational modification, specifically O‐GlcNAcylation, of Ca^2+^/CaMKII. This modification causes CaMKII to exist in a chronically upregulated state in diabetes (Daniels et al., 2018; Erickson et al., 2013; Hegyi et al., 2019) (see Table 3), causing a number of cascading effects, which we discuss below. Importantly even if glucose is later corrected, severe early hyperglycaemia can leave partially irreversible adverse effects, such as CaMKII oxidation at its Met281/282 site, which can persist after glucose levels stabilise (Anderson, 2015; Erickson et al., 2011; Lu et al., 2020).

One major consequence of this CaMKII hyperactivation is the CaMKII‐dependent phosphorylation of the Na^+^ channel, increasing I_NaL_ considerably. This leads to a prolongation of the action potential and increases influx of sodium ions into the cell (Wagner et al., 2006). This, in turn, can promote calcium overload of the cell through two distinct mechanisms. First prolonged APD leads to longer opening of L‐type calcium channels, the main source of calcium influx. Second higher sodium loading increases influx and reduces calcium efflux via I_NaCa_, increasing [Ca^2+^]_i_ (Despa & Bers, 2013), further reinforcing CaMKII hyperactivity.

Hyperactive CaMKII can promote early afterdepolarisations (EADs) through increased phosphorylation of L‐type calcium channels, which increases the amplitude of I_CaL_ (Bers & Morotti, 2014; Yuan & Bers, 1994). EADs are further promoted by APD prolongation following I_NaL_ hyperactivity. Hyperactive CaMKII and calcium overload further promote the formation of delayed afterdepolarisations (DADs). Indeed, CaMKII‐dependent phosphorylation sensitises ryanodine receptors (RyR) to Ca^2+^, increasing leak from the sarcoplasmic reticulum, which is supported by findings from mouse studies that noted an increase in calcium leakage from the sarcoplasmic reticulum in diabetic myocytes (Belke et al., 2004; Stolen et al., 2009). A lower sarcoplasmic reticulum Ca^2+^ load would lead to less peak Ca^2+^ release (see Table 4). The threshold of sarcoplasmic reticulum Ca^2+^ required to generate DADs is also lower in T2DM due to enhanced RyR activation (Popescu et al., 2019). In addition, decreased SERCA2 expression and impaired Ca^2+^ reuptake contribute to decreased Ca^2+^ transport back into the sarcoplasmic reticulum, further increasing diastolic [Ca^2+^]_i_ (Ashrafi et al., 2017) (see Tables 2 and 4).

The disruptions to Ca^2+^ and Na^+^ handling in T2DM resemble those present in heart failure, where the sodium–calcium exchanger (NCX) is hyperactive (Despa & Bers, 2013; Ebinger et al., 2005; Pogwizd, 2003). However, this is further exacerbated in T2DM by the increased activity of sodium‐glucose cotransporter, which provides another substantial source of Na^+^ influx (Lambert et al., 2015).

Data on the expression and activity levels of NCX itself in T2DM are controversial (see Table 2). Ashrafi et al. showed an overexpression of NCX1 mRNA expression in T2DM human cardiomyocytes (Ashrafi et al., 2017). Increased NCX activity was also observed in a mouse study of T2DM (Stolen et al., 2009). Increased NCX will lead to more Ca^2+^ efflux, possibly explaining the reduction observed in peak [Ca^2+^]_i_ transient (see Table 4). However, another study in diabetic rat hearts showed a decrease in protein expression and mRNA levels of NCX1, accompanied by a decrease in I_NaCa_ current density (Hattori et al., 2000). Further investigations on NCX function in T2DM are warranted to understand these differences.

Together, these alterations create two key yet opposing phenomena in T2DM: Ca^2+^ depletion and impaired contractility on one hand from sarcoplasmic reticulum leak and reduced sarcoplasmic reticulum Ca^2+^ load, and intracellular Ca^2+^ overload with arrhythmogenesis via EADs and DADs on the other. The prevalent trend will likely reflect the balance of the different ionic driving factors contributing to these effects, which may vary through time and with disease progression.

### PI3K/AKT signalling and I_CaL_

In contrast to the CaMKII‐induced increase in I_CaL_, other studies have reported a reduction in I_CaL_ in type 2 diabetes, possibly because of a reduced expression of CaV1.2, the key subunit involved in I_CaL_ (Pereira et al., 2006), which may be due to impaired PI3K/AKT signalling (Lu et al., 2011). Attenuated PI3K/AKT signalling is also responsible for increased I_NaL_ and action potential prolongation in diabetes (Lu et al., 2013) (see Table 1).

### Potassium currents and repolarisation abnormalities

Studies reported a reduction in I_to_ in rodent models of T2DM, a likely contributor of the increased APD and delayed repolarisation noted in T2DM (Magyar et al., 1992; Sato et al., 2014; Shimoni et al., 2004; Tsuchida & Watajima, 1997; Tsuchida et al., 1994). Regional differences in I_to_ reduction have also been proposed, with a greater attenuation in epicardial cells than in endocardial cells in T2DM (Sato et al., 2014; Shimoni et al., 1995). Hyperglycaemia is thought to increase angiotensin II (AngII) in tissue, including the myocardium (Giacchetti et al., 2005). AngII inhibits I_to_ via downregulation of Kv4.3 and the type 2 AngII receptor (AT2R) (Caballero et al., 2004; T.‐T. Zhang et al., 2001). AngII receptor blocker valsartan reversed the reduced I_to_ observed in a mouse model of T2DM (Shimoni, 2001), supporting the involvement of AngII in I_to_ reduction in T2DM. In addition, interleukin 1β (IL‐1β) and tumour necrosis factor α (TNF‐α) are two pro‐inflammatory cytokines that are elevated in T2DM and known to inhibit I_to_ (Zayas‐Arrabal et al., 2021).

It is important to note that although I_to_ is a key repolarising current in small rodents, it plays a comparatively much smaller role in human‐like species, which rely predominantly on I_Kr_ and to a lesser extent on I_Ks_ for repolarisation (Joukar, 2021). Thus, when changes in APD are reported in small rodents, these may be driven by changes in I_to_, and may not directly translate into similar changes in human as they are mechanistically distinct. Much less evidence is available for potassium currents most relevant for human APD control. The human gene expression study by Ashrafi et al. suggested that the ERG gene is downregulated, which causes a reduction in I_Kr_ density (see Table 1), another factor that may explain the QT prolongation and increased risk of EAD formation observed in T2DM.

## Cardiac effects of pharmacological therapies in type 2 diabetes

**Figure 3:**
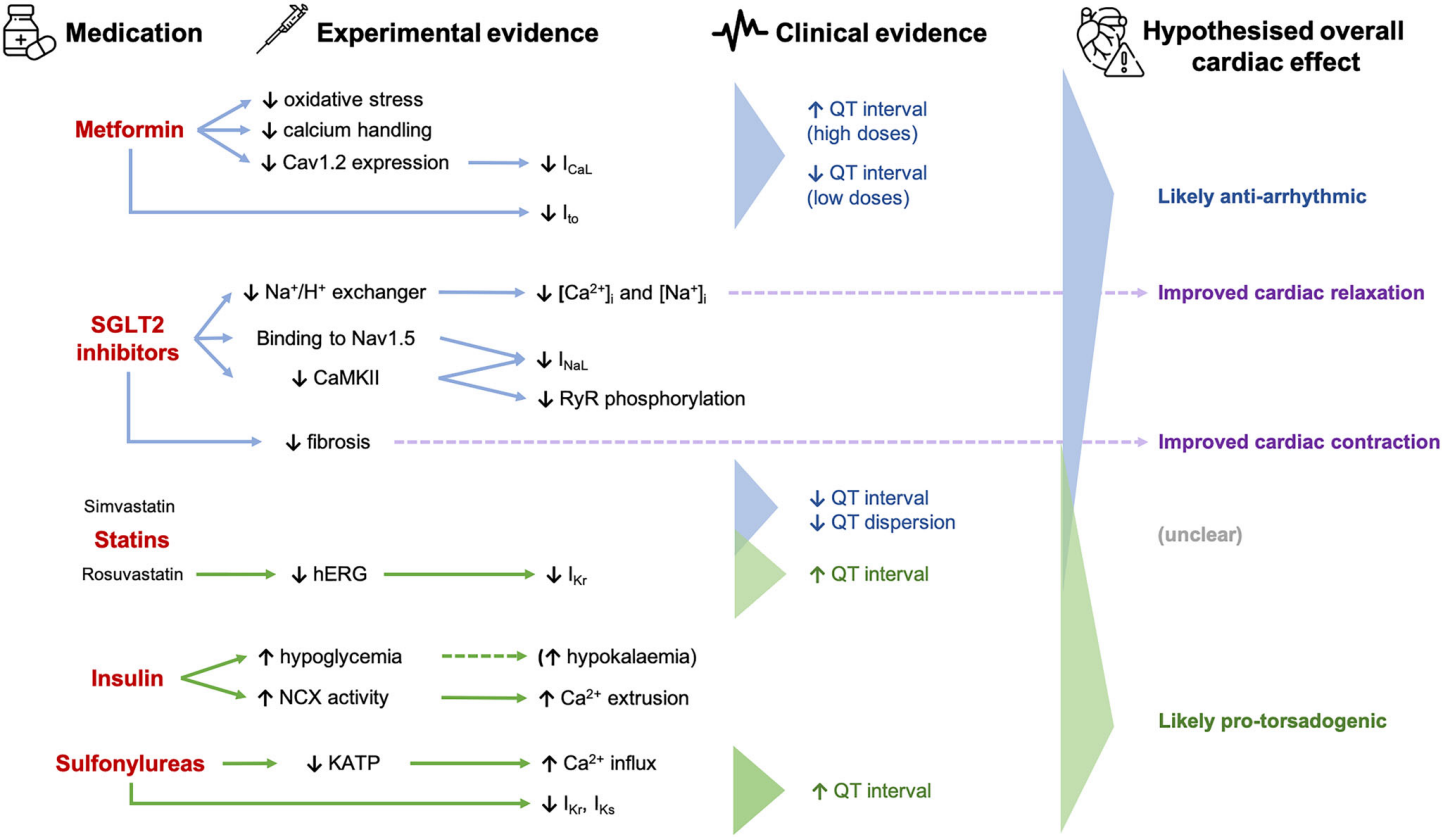
Summary of cardiac effects of common pharmacological agents taken by patients with type 2 diabetes, including glucose‐lowering medications and statins Experimental and clinical evidence supporting those effects is illustrated, along with the hypothesised overall cardiac effect of each drug category. Effects will almost certainly vary across individuals, as other important factors, such as patient age, sex, body composition, comorbidities, other medications and so forth, will also play a role in the extent of these medications’ effects on the heart of a given patient.

As demonstrated so far, the combination of ionic, fibrotic and nerve‐related cardiac remodelling driven by T2DM makes the diabetic myocardium a high‐risk substrate for cardiac arrhythmias. Pro‐arrhythmic pharmacotherapy may contribute to the increased incidence of SCD in T2DM (Lynge et al., 2020), while other medications can also have an effect on the heart's contractile function. Therefore, evaluating the cardiac effects of pharmacological agents commonly administered to T2DM patients is particularly important for patient risk stratification. In this section, we discuss the implications of glucose‐lowering drugs and other common medication in T2DM on arrhythmic risk and myocardial contractility. Key points are summarised in Fig. 3.

### Metformin

Metformin is the most common drug administered to T2DM patients. It helps lower blood glucose levels by reducing glucose production in the liver and by increasing GLP‐1 secretion and glucose utilisation in the gut (Rena et al., 2017). Experimental and clinical observational findings suggest that metformin may exert anti‐arrhythmic effects, notably by reducing atrial fibrillation risk, whereas evidence regarding its association with ventricular arrhythmias is limited (Mascarenhas et al., 2024). Possible mechanisms proposed include the attenuation of inflammation and oxidative stress, improvement in impaired calcium handling and reversal of structural myocardial remodelling (Mascarenhas et al., 2024). One study in rats reported QT interval prolongation following high doses of metformin and QT shortening at low doses (Costa et al., 2008), whereas a mouse study reported a shortening of the QT interval (Wang et al., 2020). The latter suggested that this change occurred because of a decrease in I_CaL_ due to a decrease in expression of Cav1.2 channels and its associated CACNA1C mRNA (H. Wang et al., 2020). Meanwhile, another rat study showed no change in I_CaL_ after metformin treatment, however a reduction in peak I_to_ density was found along with a prolongation of the QTc interval (Malagueta‐Vieira et al., 2022). These results may be specific to small rodents, which rely on I_to_ for APD. Furthermore, the diabetic status of the animal models was not consistent across these studies, which may contribute to the discrepancies in these results. Overall, these experimental and clinical findings motivate the need for further studies to investigate possible anti‐arrhythmic properties of metformin.

### Insulin

Although patients with type 1 diabetes are fully dependent on insulin treatment, some patients with T2DM may also be prescribed insulin therapy if other medications are insufficient to control blood glucose. Patients treated with insulin were shown to be at higher risk of developing atrial fibrillation (Chen et al., 2017; Liou et al., 2018). Insulin can induce hypoglycaemia, which can in turn trigger arrhythmias, likely due to hypokalaemia that tends to accompany hypoglycaemia (Leak & Starr, 1962). One study demonstrated a positive effect of insulin on NCX, with increased activity and expression, facilitating calcium removal and promoting better cardiac relaxation (Gök et al., 2022). In the context of decreased NCX in T2DM, this effect may have beneficial effects on calcium handling in T2DM.

### SGLT2 inhibitors

Sodium‐glucose cotransporter‐2 inhibitors (SGLT2i) are commonly administered to T2DM patients to help control blood sugar levels by preventing glucose from being reabsorbed into the kidneys (American Diabetes Association Professional Practice Committee, 2022). To date, SGLT2i are the only medication type found to have a significant proven benefit in patients with HFpEF (Anker et al., 2021; Solomon et al., 2021). Consistent findings suggest that SGLT2i reduce the risk of ventricular arrhythmias, atrial fibrillation and SCD (Curtain et al., 2021; Fernandes et al., 2021; Liao et al., 2024; Scheen, 2022). This may be, in part, due to the inhibition of Na^+^/H^+^ exchanger by SGLT2i empagliflozin, canagliflozin and dapagliflozin, resulting in reduced cytoplasmic Na^+^ and Ca^2+^ and increased mitochondrial Ca^2+^, which in T2DM may mitigate some of the pro‐arrhythmic consequences of increased [Na^+^]_i_ (Uthman et al., 2018). Myocardial expression of the SGLT1 protein is upregulated in T2DM and HFpEF; this upregulation increases [Na^+^]_i_, which in turn increases cytosolic Ca^2+^ via NCX (Banerjee et al., 2009). One study showed that the treatment of human‐induced pluripotent stem cells (hiPSCs) under high‐glucose conditions with empagliflozin restored SGLT1 expression to control levels, thus abolishing its adverse effects on increased [Na^+^]_i_ (Ng et al., 2018). Another study showed that empagliflozin inhibits the late sodium current I_NaL_ by binding directly to the Nav1.5 sodium channel (Philippaert et al., 2021), whereas Hegyi et al. suggested that I_NaL_ regulation by empagliflozin may occur via an indirect mechanism involving CaMKII inhibition (Hegyi et al., 2022). The possible involvement of CaMKII in the mechanism of action of SGLT2i is supported by another study showing that, in a mouse model of T2DM, empagliflozin inhibited CaMKII‐dependent RyR phosphorylation and reduced O‐GlcNAc levels; two key contributing factors to the pro‐arrhythmic setting in T2DM (Kadosaka et al., 2023). In addition to their effects on the heart's cellular electrophysiology, SGLT2i have been shown to reduce adverse structural remodelling such as fibrosis, as well as stabilise impaired myocardial energetics, which may further explain their direct benefits on the heart in terms of preventing arrhythmias and improving contraction (Kang et al., 2020; Lee et al., 2019; Santos‐Gallego et al., 2019).

### Sulfonylureas

Sulfonylureas, which increase plasma insulin concentration, are typically the second‐line T2DM treatment in cases where metformin fails (Tomlinson et al., 2022). Sulfonylureas stimulate insulin secretion by binding to the KATP channel in pancreatic beta cells. Their KATP channel‐blocking action also applies to cardiomyocytes, leading to membrane depolarisation, subsequent influx of Ca^2+^, APD prolongation and increased QT interval in the ECG (Najeed et al., 2002). Glibenclamide, a type of sulfonylurea, was shown to block I_Kr_ and I_Ks_, the latter to a lesser extent; this may further contribute to QT prolongation observed in patients treated with this drug (Rosati et al., 1998). Their arrhythmogenic effect, however, is complex and debated, and likely dependent on the underlying structural substrate, including the presence of myocardial ischaemia (Leonard et al., 2017). Some studies have shown an association of sulfonylureas with a higher risk of atrial fibrillation, ventricular arrhythmias and SCD compared to metformin (Islam et al., 2023; Lee et al., 2022; Zhou et al., 2022), whereas other studies have reported an anti‐arrhythmic effect of sulfonylureas, albeit mostly in ischaemic and heart failure cases (Aronson et al., 2003; Lomuscio et al., 1994; Pogátsa et al., 1988).

### Statins

Statins are commonly prescribed to patients with dyslipidaemia, which is common in T2DM, to help reduce cholesterol and triglyceride levels. Studies have shown that certain statins reduce the QTc interval, including in heart failure patients treated with atorvastatin (Vrtovec et al., 2005; Xie et al., 2010) and in heart transplant patients treated with either pravastatin or a combination of statins (Vrtovec et al., 2006). One study found a significant decrease in QT dispersion in patients with T2DM treated with simvastatin, suggesting positive effects of the drug on cardiac repolarisation heterogeneity (Tekin et al., 2008). Another study based on experimental and clinical data found an association between rosuvastatin and QT prolongation (Koo et al., 2023). These findings align with a previous study showing the hERG‐blocking effect of rosuvastatin, thus reducing I_Kr_ and prolonging cardiac repolarisation (Plante et al., 2012). These heterogeneous results warrant careful consideration when modelling the cardiac effect of statins in T2DM, as their effects seem to vary across different compounds within this drug class.

## Bridging scales: human‐based, multiscale computational modelling and simulation of the diabetic heart

Clinically, diabetic myocardial disorder remains a highly complex and multifactorial condition, making it challenging to study accurately and holistically across different biophysical scales. The mechanistic understanding of ionic and structural remodelling in T2DM obtained through experimental results in humans and animals provides a foundation for hypothesis‐driven computational exploration. However, animal models of T2DM present interspecies differences such as the balance of repolarising currents or subcellular signalling (Gallego et al., 2021). This poses a significant barrier for their translation to benefit patients. Multiscale human‐based cardiac models can help overcome this barrier by integrating experimentally observed changes, such as reduced potassium currents, CaMKII overactivation, altered I_NaL_, reduced SERCA flux and heterogeneous fibrosis, into a human‐specific virtual framework to simulate their combined effects across scales, e.g. on cellular excitability, calcium dynamics and tissue conduction. Such integrative simulations enable the quantification of how specific ionic or structural perturbations contribute to arrhythmia vulnerability or impaired contractility, and help identify which mechanisms are most arrhythmogenic in human‐like settings. Furthermore, *in silico* population modelling can incorporate patient variability in electrophysiological parameters, supporting virtual patient stratification to identify subgroups at the highest risk of arrhythmia or diastolic dysfunction. These approaches can also inform therapeutic targeting, for example, by predicting the anti‐arrhythmic efficacy of treatments specifically under diabetic conditions.

**Figure 4:**
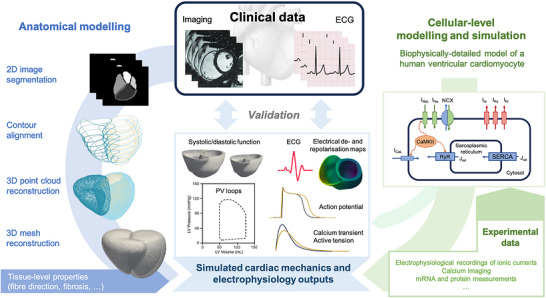
Overview of a multiscale computational modelling and simulation framework for cardiac electrophysiology and mechanics Imaging data, such as magnetic resonance imaging, are used to reconstruct the cardiac anatomy virtually in 3D. Tissue properties such as fibre direction and fibrosis can be incorporated using methods described in the main text. Functionality is embedded into these anatomical models using cellular‐level models extensively calibrated and validated on human experimental data to simulate the behaviour of the heart across multiple scales. The considerable potential of this framework in T2DM‐specific cardiac research is discussed in detail in the main text. Simulated biomarkers can then be compared against clinical data to validate the models. PV, pressure‐volume.

In this final section, we present novel human‐based computational modelling and simulation approaches to help address these challenges, offering a multiscale approach to unravel disease mechanisms and enable *in silico* trials for drug safety and efficacy assessment in T2DM, thus bridging the gap between molecular mechanisms and clinical intervention. Figure 4 provides a visual overview of the multiscale cardiac computational modelling and simulation framework, whose components we discuss in more detail below.

### Modelling and simulating human cardiac electrophysiology and contractility at cellular level

Over the past two decades, synergistic iterations between experimental science and modelling and simulation have led to the development of several cardiac biophysical models, with most recent ones from Tomek et al. (Tomek et al., 2019, 2025) and Bartolucci et al. (Bartolucci et al., 2020), building on work by Ten Tusscher and Panfilov, Grandi et al., O'Hara et al. for human ventricular electrophysiology and excitation–contraction coupling (Grandi et al., 2010; T. O'Hara et al., 2011; ten Tusscher & Panfilov, 2006), β‐adrenergic stimulation (Heijman et al., 2011), contractility (Coppini et al., 2019; Land et al., 2017) and coupled electromechanics (Margara et al., 2021). Largely based on Hodgkin‐Huxley equations and Markov models, detailed mathematical representations of cardiac channels, pumps, exchangers and ion handling processes allow these models to accurately simulate cardiac action potentials, calcium transients and active tension dynamics under healthy and disease conditions, as well as drug block. Importantly, independent validation against datasets not used in model development and calibration is increasingly carried out, such as in ToR‐ORd or T‐World models (Tomek et al., 2019, 2025). This demonstrates that such models are coherent and predictive even outside the primary domain of application, making them useful tools to predict and explain the impact of perturbation by drugs or disease. By adapting these models for T2DM, we can recapitulate key cellular characteristics of the disease and use the model as a testbed for further investigations across different scales.

An early computational cellular‐level model of T2DM was developed by Ashrafi et al. based on mRNA data (Ashrafi et al., 2017), however it showed a strongly exaggerated phenotype, including extreme APD prolongation and virtually abolished calcium transient. Recently, Tomek et al. explored a range of possible T2DM phenotypes using a novel model, T‐World, which incorporates human data and available data in animals (Tomek et al., 2025). They reported consistently increased risk of EADs and alternans, which contribute to elevated risk of SCD in T2DM. Interestingly, the elevated risk of EADs persisted across models of T2DM with both increased and decreased I_CaL_, the central driver of EAD formation. The elevated EAD risk arises mainly from the combined effects of other factors, such as reduced I_Kr_ and increased I_NaL_ and I_NaCa_, rather than from a single change alone.

The elevated EAD risk may also explain the high proportion of T2DM patients dying of SCD with potentially pro‐arrhythmic medication (Lynge et al., 2020). The implication of those findings is that drug dosage that is safe for normal patients may be riskier in patients with T2DM, warranting a disease‐specific approach to the prescription of potentially pro‐arrhythmic drugs. That said, given the heterogeneous data sources, this study highlights the need for collection of larger‐scale human cardiac data on the T2DM phenotype, particularly protein expression to capture true functional (not just transcriptional) changes.

One of the key advantages of computer models is their ability to integrate and explain interconnected behaviours while also clarifying the mechanisms that link them. For example, clinical data show that patients with T2DM with diastolic dysfunction have an increased risk of alternans. Tomek et al. utilised computational modelling to show that the relationship can be explained by variation in levels in SERCA pumps (Tomek et al., 2025), indicating that the degree of diastolic dysfunction could correlate with arrhythmia risk, though alternans is clearly not the sole pro‐arrhythmic factor in T2DM. Diastolic dysfunction can arise from multiple causes, suggesting that T2DM patients prone to alternans may respond better to therapies aimed at restoring SERCA levels than to other treatments that primarily address other mechanisms.

### Modelling cardiac anatomy in 3D

Based on whole‐organ images acquired using high‐resolution clinical imaging modalities like CMR or computed tomography, it is possible to create a virtual and physiologically accurate representation of the patient's cardiac anatomy in 3D. These reconstruction pipelines typically rely on convolutional neural networks and/or statistical shape models to segment the images, delineate the myocardium and obtain a smooth surface representing the heart in 3D (Bai et al., 2018; Banerjee et al., 2021, 2022; Chen et al., 2020; Sander et al., 2023; Strocchi et al., 2024). These surfaces can be transformed further into tetrahedral meshes, including rule‐based cardiac fibre orientation (Doste et al., 2019; Labarthe et al., 2014; Strocchi et al., 2024; Villard et al., 2018). These patient‐specific virtual cardiac anatomies form the basis of whole‐organ cardiac electrophysiological and electromechanical simulations in 3D, which we discuss below.

In an earlier section, we described cardiac structural and anatomical changes that occur in T2DM. Recent modelling work has proposed a unified framework linking left ventricular geometry and tissue properties with pressure‐volume relations, enabling analysis of cardiac function both under physiological loading and from purely geometric data (Zhang et al., 2023). Such frameworks could help bridge anatomical remodelling in T2DM with biomechanical and haemodynamic function.

### Modelling cardiac fibrosis

Advanced imaging techniques such as late gadolinium‐enhanced (LGE) CMR and T1 mapping are essential for characterising myocardial fibrosis non‐invasively in T2DM (Ng et al., 2012; Zhang et al., 2019), with T1 mapping‐derived extracellular volume fraction serving as a proxy marker of diffuse fibrosis (Haaf et al., 2016). O'Hara et al. used fused LGE‐T1 CMR data from human hypertrophic cardiomyopathy patients and threshold‐based rules to obtain a personalised reconstruction of focal and diffuse fibrosis in a virtual heart built from the same imaging data (O'Hara et al., 2022). Another computational study investigated the pro‐arrhythmic effects of adipose and fibrotic tissue in the heart in 2D using the TP06 model of a ventricular cardiomyocyte; fibrosis was modelled as diffuse, with non‐conductive nodes randomly interspersed throughout the 2D grid (De Coster et al., 2018). Further mathematical concepts such as Perlin noise have been applied to simulate fibrosis in 3D, generating physiologically realistic, virtual tissue‐level representations of different types of fibrosis with automatic calibration based on histological image slices (Lawson et al., 2024). Coleman et al. incorporated the effects of diffuse fibrosis on electrical conduction in a virtual whole‐organ study by modelling delayed electrical wave propagation by stochastically assigning certain elements in the cardiac mesh as unexcitable using a probability density function (Coleman et al., 2024). Another study looking at arrhythmogenesis in non‐ischaemic cardiomyopathy used an efficient discrete finite element framework to model fibrosis as infinitesimal splits in the mesh, capturing complex fibrosis networks (Balaban et al., 2020). This method reproduced re‐entrant electrical activity and conduction slowing at high pacing rates, demonstrating that fibrosis density and topology critically influence transient conduction block and re‐entry. These personalised reconstruction approaches coupled with mathematical modelling are highly promising avenues for computational studies of the effects of myocardial fibrosis and arrhythmic risk in T2DM.

In the context of electromechanical modelling, the effects of fibrosis on myocardial stiffness must be accounted for. A simple approach to achieve this is to model fibrosis as a global and isotropic increase in tissue stiffness, reflecting the elevated collagen content in the extracellular matrix leading to restricted inflation of the ventricles. A more advanced approach was proposed by Wang et al. to account for spatial tissue heterogeneity and mechanical anisotropy by harnessing high‐resolution confocal imaging of cardiac tissue to identify collagen organisation in the perimysium and endomysium and model direction‐dependent mechanical stiffening (Wang et al., 2016). Alternatively,w stiffness properties could be varied locally in the mesh once fibrotic regions have been generated using methods described above (Balaban et al., 2020; Coleman et al., 2024; De Coster et al., 2018; Lawson et al., 2024).

### Modelling and simulating electrophysiological and electromechanical behaviour across scales

Tissue‐level electrophysiology is simulated using the bidomain model for cardiac conduction (Tung, 1978), with the simplified monodomain model offering computational efficiency and minimal accuracy loss (Nielsen et al., 2007; Potse et al., 2006). This model assumes a linear relationship between intracellular and extracellular domains described by a reaction–diffusion equation. Numerical schemes such as the finite element and volume methods discretise space, while time discretisation is handled by forward and backward Euler methods (Niederer et al., 2011; Vergara et al., 2016). An approximation to the solution of the discretised equations is then obtained using software MonoAlg3D with high‐performance parallel computing, yielding simulated electrical propagation and repolarisation from ionic dynamics to the ECG (Gima & Rudy, 2002; Pezzuto et al., 2017; Potse, 2018).

This approach has been used extensively to model cardiac electrical propagation and repolarisation for *in silico* studies of the heart in different disease and therapeutic contexts, as introduced earlier. However, T2DM remains a relatively unexplored clinical application area of these methods. Sedova et al. carried out a whole‐organ electrophysiological simulation study of diabetes to determine how ventricular repolarisation dispersion and repolarisation gradients across orthogonal directions in the ventricles contribute to the formation of T‐wave flattening (Sedova et al., 2017). However, this study reproduced behaviours observed in a rabbit model of diabetes type 1, not 2. Furthermore, ventricular repolarisation was modelled as a single dipole located in the centre of the ventricles and generating a potential distribution, instead of simulating electrical propagation using a truly multiscale approach such as the monodomain equation coupled with accurate ionic current representations. Recently, Bertrand et al. proposed a biophysically detailed, multiscale computational framework of the human heart under T2DM conditions (Bertrand et al., 2025). They integrated experimental data‐based cellular‐level changes, described above, into a human cardiomyocyte model, embedded in a patient‐specific 3D cardiac anatomy reconstruction with personalised tissue conduction speeds and local electrical activation properties. This study represents a first step towards *in silico* investigations of T2DM‐specific cardiac electrophysiology across different biophysical scales. Another relevant computational study modelled the intrinsic cardiac autonomic nervous system, including cardiac ganglia and sympathetic nerve activity in the atria, to investigate its effect on the initiation and maintenance of atrial fibrillation (Hwang et al., 2017). Computational modelling of cardiac innervation in the ventricles, however, remains unexplored, but it represents a very promising avenue to explore in T2DM, given the underlying pathophysiological mechanisms discussed earlier. The cardiac conduction system has also been modelled successfully *in silico*, accurately replicating the intricate branching patterns of Purkinje fibres observed in macroscopic histological studies of the heart and capturing electrophysiological heterogeneities at tissue level (Berg et al., 2023; Deo et al., 2009; Sahli Costabal et al., 2016; Sebastian et al., 2013). These models can be further personalised using optimisation‐based methods to generate patient‐specific Purkinje fibre networks that best fit an individual's given cardiac anatomy and reproduce realistic activation timings (Berg et al., 2023). Together these methods represent exciting avenues to model the electrophysiological abnormalities and tissue‐level heterogeneities that are present in the T2DM myocardium, and to quantify the impact of these abnormalities, relative to ionic remodelling, at organ level.

Whole‐organ modelling of cardiac electromechanical function has successfully enabled *in silico* characterisation of the heart's mechanical properties, both under healthy and disease conditions. Strocchi et al. developed a four‐chamber model of the heart to simulate ventricular contraction with a focus on the mechanics of the pericardium (Strocchi, Gsell, et al., 2020). A number of such four‐chamber models for electromechanical simulations were made publicly available, enabling future large‐scale studies (Strocchi, Augustin, et al., 2020). Wang et al. presented a multiscale electromechanical simulation framework to assess cardiac function in a postinfarction setting (Wang et al., 2021). By incorporating membrane kinetics excitation–contraction and active tension cellular models (Levrero‐Florencio et al., 2020; Margara et al., 2021), and representing ischaemic electromechanical remodelling at tissue level, Wang et al. were able to recapitulate key mechanical consequences of myocardial stiffness, including changes in diastolic and systolic function, by simulating the behaviour of the ventricles in 3D (Wang et al., 2021). Simulations were achieved using a high‐performance software, Alya, developed for coupled multiphysics and multiscale problems (Santiago et al., 2018). Recently, Strocchi et al. proposed a multiscale, whole‐heart electromechanics framework for (all‐type) diabetes, integrating ventricular and atrial cellular‐level models, anatomically detailed whole‐heart mechanics and a 0D circulatory system model (Strocchi, Barrows, et al., 2025). They did not explicitly account for diabetes‐specific ionic current changes but rather focused on the sensitivity of physiological parameters across scales to identify key determinants of cardiac mechanical dysfunction in diabetes, which, they found, included systemic resistance, venous pressure and atrial stiffness.

Subject to the availability of data that are robust enough for accurate, personalised measurements, e.g. ischaemic scar using LGE or interstitial fibrotic patches using T1 mapping, such frameworks may be harnessed to carry out further *in silico* investigations of cardiac electrophysiology and mechanics in T2DM. Although this review focuses primarily on structural, electrophysiological and electromechanical modelling, T2DM also affects the circulatory system and vascular haemodynamics. Recent work by Tunedal et al. combined 4D flow magnetic resonance imaging data with subject‐specific mathematical models of central haemodynamics to investigate haemodynamic alterations in T2DM and hypertension, highlighting impaired left ventricular relaxation (Tunedal et al., 2023). Future developments may extend current electrophysiological and electromechanical frameworks, described above, to include vascular and haemodynamic components, enabling a more comprehensive representation of the multiscale effects of T2DM on the wider cardiovascular system.

### Modelling and simulating sex‐specific differences

One clinical study suggested that T2DM may induce sex‐specific patterns of myocardial remodelling, with female patients exhibiting concentric left ventricular remodelling to a further extent, whereas male patients showed more‐pronounced extracellular matrix remodelling and subclinical diastolic dysfunction (Shang et al., 2022). In an experimental study using mice, t diabetic female cardiomyocytes exhibited sex‐specific alterations in gene and protein expression, affecting cytoskeletal organization, hypertrophy and calcium handling, including SERCA and excitation–contraction coupling, which differs from male counterparts (Harper et al., 2024). These differences may reflect divergent pathophysiology of diabetic cardiomyopathy between sexes and may contribute to the well‐established sex‐specific differences in disease trajectory, notably regarding the development of heart failure with reduced ejection fraction, which is typically more common in men, *vs*. preserved ejection fraction, typically more common in women (Beale et al., 2018; Campbell et al., 2024; Lam et al., 2019).

Cardiac modelling and simulation frameworks allow us to quantify sex‐specific differences by incorporating computational models of human male and female cardiomyocytes and by harnessing patient‐, and therefore sex‐specific, 3D cardiac anatomy reconstructions. Yang and Clancy proposed male‐ and female‐specific ventricular cardiomyocyte models incorporating genomic differences and sex steroid effects to predict arrhythmic risk (Yang & Clancy, 2012), while another computational study harnessed these models to generate synthetic data to build sex‐specific machine learning‐based classifiers for drug‐induced Torsade de Pointes risk (Fogli Iseppe et al., 2021). More recently, Holmes et al. calibrated sex‐specific human cardiomyocyte models with protein and genomic expression data and embedded these models into biventricular anatomical reconstructions for multiscale human‐based simulations, examining ionic and organ‐level mechanisms underlying sex differences in repolarisation, arrhythmogenesis and drug responses (Holmes et al., 2025). Subject to further sex‐specific human T2DM data these approaches could be incorporated into the T2DM‐specific frameworks presented earlier, expanding their potential scope and helping to overcome inherent sex‐related limitations of experimental (lack of widespread use of female animal models) and clinical research (historical underrepresentation of women in clinical studies).

### *In silico* evaluation of drug safety and efficacy

To date, most of the information on the effectiveness and safety of drugs in pre‐clinical stages is derived from animal models. Although beneficial, there are challenges in the translation of these findings to humans, including mechanistic relevance, as pathophysiological mechanisms may differ between species and sex‐specific effects, as experimental models often include male animals only. Moreover, while animal models are sometimes viewed as focusing on single variables, in reality they cannot isolate one independent factor entirely, since biological systems include intrinsic feedback mechanisms that compensate for perturbations and confound direct causal inference. In contrast, computational models can selectively control or disable these feedback loops, allowing us to explore the effect of truly independent variables.

*In silico* trials provide an alternative pathway to evaluate drug safety and efficacy by simulating the action of drug in a highly controllable virtual human environment instead of a live animal model (Viceconti et al., 2021). Using a multiscale framework similar to that of disease remodelling, the known or hypothesised mechanistic action of a certain pharmacological compound is modelled at intracellular level, and its downstream effects are examined by quantifying changes in simulated electrophysiological or electromechanical biomarkers at cellular and/or whole‐organ level. This approach has been applied in various disease contexts, successfully incorporating cellular remodelling into pharmacological simulations, demonstrating the feasibility of this approach (Dasí et al., 2022; de Oliveira & Niederer, 2016; Mirams et al., 2014; Passini et al., 2017; Tomek et al., 2019; Wang et al., 2025). This novel computational approach remains tightly intertwined with experimental studies, which are crucial in providing data that quantify the effects of a given compound on protein expression and/or ionic current density.

Contingent on the availability of sufficient and reliable experimental data, and on the extensive validation of virtual T2DM cardiac models, *in silico* drug evaluation holds huge potential to investigate the multiscale cardiac effects of drugs in T2DM. As discussed in previous sections, this could include evaluating candidate therapies such as I_NaL_ and CaMKII inhibition within anatomically detailed, disease‐specific models. Such an approach would be especially valuable in high‐risk clinical scenarios that have been poorly studied so far, such as the complex overlap of T2DM, HFpEF and ventricular arrhythmias, while recognising that robust data and further model validation remain key challenges.

### Limitations

As with any modelling approach, computational models of cardiac cells have inherent limitations that must be considered, particularly when representing complex conditions such as T2DM. Computer models can only simulate proteins, pathways and processes explicitly incorporated into their structure. Although current models are increasingly comprehensive and capture a wide spectrum of physiological phenomena (Tomek et al., 2025), they remain tools including an incomplete representation of biological reality. Many signalling pathways or regulatory mechanisms are not included yet, which is why some mechanisms contributing to the overall disease phenotype may be overlooked. Despite these limitations, computational models are highly valuable as tools to probe pathophysiological systems, such as diabetes, when used synergistically with experimental approaches. Their strength lies in the mechanistic and interpretable nature of their framework: they explicitly represent known biological processes such as ion channel kinetics or intracellular signalling cascades. This structure allows for the direct integration of experimentally observed cellular remodelling into simulations, as well as for systematic validation of computational predictions against experimental and literature data. Thus, when combined with animal or *in vitro* studies, computer models contribute to a more comprehensive understanding of the mechanisms underlying pathological remodelling in cardiovascular disease than either approach can provide alone.

Ultimately, no model can (and should not) fully capture the complexity of biological systems. Careful selection or development of a model that is fit for purpose, while remaining mindful of its assumptions and limitations, is essential for rigorous and meaningful application of computational modelling and simulation in cardiac research.

## Conclusion

In this review, we explored key mechanisms that underpin the adverse effects of T2DM on the heart at different scales. Subcellular disturbances involving CaMKII, impaired calcium handling and ionic current remodelling, coupled with diffuse myocardial fibrosis, act as an arrhythmic trigger and substrate, respectively, while causing impaired contractility, and diastolic and systolic dysfunction. Damage to the intrinsic cardiac nervous system can act as a further trigger and substrate for ventricular arrhythmias in T2DM. We also reviewed evidence on the cardiac effects of pharmacological therapies in T2DM, notably in terms of arrhythmic risk and contractility. We then presented relevant human‐based, multiscale computational modelling and simulation approaches that hold potential to reproduce pathophysiological mechanisms and simulate the behaviour of the diabetic heart *in silico*, complementing experimental findings by providing quantitative insights into the underlying mechanisms and consequences of T2DM‐induced cardiac abnormalities. These frameworks also open novel research avenues for *in silico* therapy evaluation in T2DM. Thus, subject to sufficient and satisfactory data availability and quality, computational modelling and simulation can enable a more efficient clinical translation of basic scientific developments and help reduce the use of animal models in pre‐clinical settings in T2DM. Ultimately, these novel methods represent a promising approach to improve our mechanistic understanding of the cardiac implications of T2DM, provide more personalised diagnosis and patient risk stratification, and enable optimal therapy planning to improve patient outcomes.

## Additional information

### Competing interests

All authors declare that they have no conflicts of interest.

### Author contributions

A.B. and J.T. conceived and designed the work; drafted the manuscript; critically revised the manuscript; conception and preparation of the figures; final approval of the version to be published; agreement to be accountable for all aspects of the work. B.R. conceived and designed the work; critically revised the manuscript; final approval of the version to be published; agreement to be accountable for all aspects of the work.

### Funding

This work was supported by an Engineering and Physical Sciences Research Council (EPSRC) Centre for Doctoral Training in Health Data Science Scholarship (EP/S02428X/1) (A.B.), a Sir Henry Wellcome Fellowship (222781/Z/21/Z) (J.T.), a Wellcome Trust Fellowship in Basic Biomedical Sciences (214290/Z/18/Z) (B.R.) and the EPSRC project CompBioMed X (EP/X019446/1) (B.R.).

## Supporting information


Peer Review History
