# Supplementary material for: Cardiac remodelling in type 2 diabetes: Pathophysiological mechanisms and opportunities for multiscale computational modelling and simulation
Source: J Physiol. 2026 Jan 2;604(13):5170–99. doi: 10.1113/JP287140 (PMC13327775; doi:10.1113/JP287140)
Supplement: Supplementary file 1 — Peer Review History [file TJP-604-5170-s001.pdf]

The Journal of Physiology

Cardiac remodelling in type 2 diabetes: pathophysiological mechanisms and opportunities for multi-scale computational modelling and simulation

Ambre Bertrand, Jakub Tomek, and Blanca Rodriguez
DOI: 10.1113/JP287140

Corresponding author(s): Ambre Bertrand (ambrebertrand14@gmail.com)

The following individual(s) involved in review of this submission have agreed to reveal their identity: Yingnan Zhang (Referee #1); Åshild Telle (Referee #2)

Review Timeline:

Submission Date:	29-Jul-2025
Editorial Decision:	08-Sep-2025
Revision Received:	14-Nov-2025
Accepted:	01-Dec-2025

Senior Editor: Laura Bennet

Reviewing Editor: Viviane Timmermann

Transaction Report:

(Note: With the exception of the correction of typographical or spelling errors that could be a source of ambiguity, letters and reports are not edited. Depending on transfer agreements, referee reports obtained elsewhere may or may not be included in this compilation. Referee reports are anonymous unless the Referee chooses to sign their reports.)

Dear Dr Bertrand,

Re: JP-TR-2025-287140 "Cardiac remodelling in type 2 diabetes: pathophysiological mechanisms and opportunities for computational multi-scale modelling and simulation" by Ambre Bertrand, Jakub Tomek, and Blanca Rodriguez

Thank you for submitting your manuscript to The Journal of Physiology. It has been assessed by a Reviewing Editor and by 2 expert referees and we are pleased to tell you that it is potentially acceptable for publication following satisfactory major revision.

The referee reports are copied at the end of this email.
Please address all the points raised and incorporate all requested revisions or explain in your Response to Referees why a change has not been made. We hope you will find the comments helpful and that you will be able to return your revised manuscript within 2 months. If you require longer than this, please contact journal staff: jp@physoc.org. Please note that this letter does not constitute a guarantee for acceptance of your revised manuscript.

ABSTRACT FIGURES: Authors are expected to use The Journal's premium BioRender account to create/redraw their Abstract Figures. Information on how to access this account is here:
https://physoc.onlinelibrary.wiley.com/journal/14697793/biorender-access.
This will enable Authors to create and download high-resolution figures. If authors have used the free BioRender service, they can use the instructions provided in the link above to download a high-resolution version suitable for publication.
The link provided should only be used for the purposes of this submission. Authors will be charged for figures created on this account if they are not related to this manuscript submission.

Your revised manuscript should be submitted online using the link in your Author Tasks: Link Not Available. This link is accessible via your account as Corresponding Author; it is not available to your co-authors. If this presents a problem, please contact journal staff (jp@physoc.org). Image files from the previous version are retained on the system. Please ensure you replace or remove any files that are being revised.
If you do not wish to submit a revised version of your manuscript, you must inform our journal staff (jp@physoc.org) or reply to this email to request withdrawal. Please note that a manuscript must be formally withdrawn from the peer review process at one journal before it may be submitted to another journal.

REVISION CHECKLIST:
Check that your Methods section conforms to journal policy: https://jp.msubmit.net/cgi-bin/main.plex?form_type=display_requirements#methods.

Check that data presented conforms to the statistics policy: https://jp.msubmit.net/cgi-bin/main.plex?form_type=display_requirements#statistics.

Upload a full Response to Referees file. To create your 'Response to Referees': copy all the reports, including any comments from the Senior and Reviewing Editors, into a Microsoft Word, or similar, file and respond to each point, using font or background colour to distinguish comments and responses and upload as the required file type.

Please upload two versions of your manuscript text: one with all relevant changes highlighted and one clean version with no changes tracked. The manuscript file should include all tables and figure legends, but each figure/graph should be uploaded as separate, high-resolution files.

You may also upload:
- 'Potential Cover Art' for consideration as the issue's cover image
- Appropriate Supporting Information (Video, audio or data set: see https://jp.msubmit.net/cgi-bin/main.plex?form_type=display_requirements#supp).

IMPORTANT POINTS TO NOTE WHEN REVISING YOUR MANUSCRIPT:
LANGUAGE EDITING AND SUPPORT FOR PUBLICATION: If you would like help with English language editing, or other article preparation support, Wiley Editing Services offers expert help, including English Language Editing, as well as translation, manuscript formatting, and figure formatting at www.wileyauthors.com/eeo/preparation. You can also find resources for Preparing Your Article for general guidance about writing and preparing your manuscript at www.wileyauthors.com/eeo/prepresources.

TRANSPARENT PEER REVIEW POLICY: To improve the transparency of its peer review process, The Journal of Physiology publishes online as supporting information the peer review history of all articles accepted for publication. Readers will have access to decision letters, including Editors' comments and referee reports, for each version of the manuscript, as well as any author responses to peer review comments. Referees can decide whether or not they wish to be named on the peer review history document.

We look forward to receiving your revised submission.
If you have any queries, please reply to this email and we will be pleased to advise.

Yours sincerely,

Laura Bennet
Senior Editor
The Journal of Physiology

REQUIRED ITEMS

- Please include an Abstract Figure file, as well as the Figure Legend text within the main article file. The Abstract Figure is a piece of artwork designed to give readers an immediate understanding of the Review Article and should summarise the main conclusions. If possible, the image should be easily 'readable' from left to right or top to bottom. It should show the physiological relevance of the Review so readers can assess the importance and content of the article. Abstract Figures should not merely recapitulate other figures in the Review. Please try to keep the diagram as simple as possible and without superfluous information that may distract from the main conclusion of the Review. Abstract Figures must be provided by authors no later than the revised manuscript stage and should be uploaded as a separate file during online submission labelled as File Type 'Abstract Figure'. Please ensure that you include the figure legend in the main article file. All Abstract Figures will be sent to a professional illustrator for redrawing and you may be asked to approve the redrawn figure before your paper is accepted.

- Your MS must include a complete "Additional information section" with the following 4 headings and content:

Competing Interests: A statement regarding competing interests. If there are no competing interests, a statement to this effect must be included. All authors should disclose any conflict of interest in accordance with journal policy.

Author contributions: Each author should take responsibility for a particular section of the study and have contributed to writing the paper. Acquisition of funding, administrative support or the collection of data alone does not justify authorship; these contributions to the study should be listed in the Acknowledgements. Additional information such as 'X and Y have contributed equally to this work' may be added as a footnote on the title page.

It must be stated that all authors approved the final version of the manuscript and that all persons designated as authors qualify for authorship, and all those who qualify for authorship are listed.

Funding: Authors must indicate all sources of funding, including grant numbers. If authors have not received funding, this must be stated.

It is the responsibility of authors funded by RCUK to adhere to their policy regarding funding sources and underlying research material. The policy requires funding information to be included within the acknowledgement section of a paper. Guidance on how to acknowledge funding information is provided by the Research Information Network. The policy also requires all research papers, if applicable, to include a statement on how any underlying research materials, such as data, samples or models, can be accessed. However, the policy does not require that the data must be made open. If there are considered to be good or compelling reasons to protect access to the data, for example commercial confidentiality or legitimate sensitivities around data derived from potentially identifiable human participants, these should be included in the statement.

Acknowledgements: Acknowledgements should be the minimum consistent with courtesy. The wording of acknowledgements of scientific assistance or advice must have been seen and approved by the persons concerned. This section should not include details of funding.

- Author profile(s) must be uploaded via the submission form. Authors should submit a short biography (no more than 100 words for one author or 150 words in total for two authors) and a portrait photograph of the two leading authors on the paper. These should be uploaded and clearly labelled together in a Word document with the revised version of the manuscript. Any standard image format for the photograph is acceptable, but the resolution should be at least 300 DPI and preferably more. A group photograph of all authors is also acceptable, providing the biography for the whole group does not exceed 150 words.

EDITOR COMMENTS

Reviewing Editor:

Reviewer 1 has rightly noted that the introduction does not clearly specify whether therapies are glucose-, HFpEF-, or arrhythmia-directed, and I support this request for clarification. Both reviewers emphasise the need for a unifying framework that links cellular, structural, and 3D modelling to pharmacological applications; I agree this would substantially strengthen the paper. As the reviewers have highlighted, the "in silico" section must be more balanced, with limitations acknowledged and supported by appropriate citations, including recent electromechanics and haemodynamic work. Both reviewers have stressed the lack of figures, and I strongly support adding visualisations, as well as integrating the tables more directly with the text. Finally, I support Reviewer 2's detailed technical suggestions (fibrosis terminology, LV volume changes, calcium handling, HRV, sex differences, and consistency issues), which will improve precision and readability.

Please also see 'Required Items' above.

REFEREE COMMENTS

Referee #1:

The manuscript provides a review of the pathophysiological mechanisms underlying electrophysiological, structural, and autonomic remodeling in type 2 diabetes (T2DM), as well as the clinical implications of these changes.

The introduction is effective in highlighting the high prevalence of T2DM and its association with increased cardiovascular risk. The authors correctly emphasize that the limited mechanistic understanding of sudden cardiac death (SCD) in T2DM poses a barrier to the development of novel therapies. However, I recommend the authors provide greater clarity regarding the therapies being referenced in this section. For instance, are these therapies aimed at glucose control, heart failure with preserved ejection fraction (HFpEF) management, or arrhythmia-specific interventions? Current HFpEF management already has well-established clinical protocols-how would the proposed novel therapies differ from or improve upon these existing approaches?

In addition, the statement "Early identification of cardiac abnormalities in T2DM is therefore crucial to mitigate risk of heart failure and arrhythmias. In the ECG, QT segment and T wave irregularities, notably QT prolongation and T wave flattening, are common in T2DM even without overt heart failure or ischemia" gives the impression that the manuscript may also be reviewing diagnostic approaches. However, this theme is not further developed in the subsequent sections. Explicitly clarifying how these points connect to the main scope of the review would demonstrate greater expertise and strengthen the translational impact of the paper.

The review's strength lies in its detailed examination of both structural and subcellular electrophysiological remodeling, with an in-depth explanation of ion channel changes related to T2DM. This section is thorough and well-referenced. The manuscript would benefit from a more explicit link between these mechanistic insights and how computational modeling could bridge current knowledge gaps, for example, by identifying new therapeutic targets or stratifying patient subgroups at highest arrhythmic risk.

The section "In silico evaluation of drug safety and efficacy" reads somewhat overconfident and lacks sufficient supporting evidence. Firstly, the authors argue that computational heart models are superior to animal models due to interspecies differences with humans. While this is an important point, the statement should be more balanced. The validation of computational heart models-particularly in the context of pharmacological development-still relies heavily on animal studies. The key advantage of computational models is that they can accelerate the exploratory phase of pharmacological discovery and are more cost-effective compared to animal experiments. Acknowledging both the strengths and the current limitations would improve the credibility of this section. Secondly, this paragraph has the potential to be one of the highlights of the review, as it directly addresses the rationale for developing a T2DM-specific cardiac model. However, the literature cited does not sufficiently connect the concept of 3D anatomical modeling with pharmacological evaluation. If a fully developed 3D heart model that incorporates T2DM-specific cellular EP properties is not yet available, the authors could strengthen the discussion by citing analogous studies in other disease contexts where cellular remodeling has been incorporated into pharmacological modeling (e.g., PMID: 39973098). This would demonstrate that the approach is feasible and underscore its potential applicability in T2DM. Overall, I recommend that the authors expand and reframe this section to more clearly emphasize how T2DM-specific computational models could contribute to drug safety and efficacy evaluation, while providing a balanced discussion of both opportunities and current challenges.

Finally, the manuscript would be strengthened by the inclusion of more visualizations. Figures illustrating structural substrates, cellular models, and examples of computational simulations would make the review more accessible and engaging for readers, especially those less familiar with the technical aspects. At present, the text is heavily descriptive, and complementary visual aids would significantly improve clarity and impact.

Referee #2:

Bertrand, Tomek, and Rodriguez present a thorough review of cardiac remodeling commonly observed in patients with type 2 diabetes. This is an important and interesting topic, given the elevated cardiovascular risks associated with diabetes. As the authors note, there are currently very few models to date focusing on these aspects, but the potential for further exploration is clear. I found the connections to interstitial fibrosis and ion channel remodeling particularly compelling.

I have some suggestions and questions - I hope they come across as constructive rather than critical.

Major:

• The emphasis on interstitial fibrosis is refreshing and, from a computational perspective, under-studied compared to replacement fibrosis. However, I was occasionally unsure about the distinctions:

◦ In the schematics, there is an arrow from "increased interstitial fibrosis" to "LGE MRI." LGE MRI best captures replacement fibrosis, while T1 imaging is more suitable for interstitial fibrosis; would it be an idea to display a T1 image instead?

◦ In the "Fibrosis" section, the authors alternate between "diffuse interstitial fibrosis" and the more general "fibrosis." Are all studies mentioned here focused on interstitial fibrosis?

◦ Similarly, in the section on computational models of fibrosis, do the studies concern interstitial fibrosis specifically, or other forms?

◦ Are all computational models discussed EP studies, or do some include mechanics? What do the authors think should differ in the representation of fibrosis in EP versus mechanics in future electromechanical 3D models?

• In the schematics, LV diastolic and systolic volumes are marked as "decreasing," while LV concentric remodeling is shown as "increasing." The text focuses on concentric remodeling. Could the authors describe the decreasing volume aspects?

◦ Does thickening combined with decreasing volume suggest inward thickening, where the blood pool decreases while the volume contained by the pericardium remains roughly the same?

◦ Is there literature relevant for comparing systolic/diastolic volume changes under pressure (giving end-diastolic and end-systolic volumes) versus pure geometrical changes (without blood flow)?

◦ Does a constant LV mass index imply an increase in both LV mass and BMI, or no change in either?

• Although not the main focus of the review, is there any anatomical remodeling of the left atrium worth mentioning? For example, thickening of the atrial walls could be relevant in the context of atrial fibrillation.

• The variance in increased vs. decreased L-type calcium channel (ICaL) conductance is interesting, as these changes would have opposite effects on both arrhythmogenicity and contractility. Do the authors have any intuition about which trend is more prevalent? Are these changes mostly affecting maximum amplitude or baseline (diastolic) levels?

• While the review focuses on ECG changes, arrhythmogenicity, and diastolic dysfunction, many of the mechanisms discussed could also contribute to systolic dysfunction. This is briefly mentioned in the context of fibrosis, but other mechanisms would also impair contractility, especially those affecting intracellular calcium. Could the authors expand on this aspect?

• I found the section about the damages to the electrical conductive system (SA/AV node and Purkinje fibers) interesting.

◦ "In the early stages of T2DM-induced cardiac autonomic neuropathy, parasympathetic function decreases relative to sympathetic function which increases, causing heart rate variability to decrease." What are the (negative) consequences of decreased heart rate variability?

◦ Is heart rate variability measured beat-to-beat in the same patient, or across different activities?

◦ What do the authors think about the relative impact of conduction system damage versus interstitial fibrosis on (1) arrhythmogenicity and (2) systolic function? Which do they consider more consequential?

• The review mentions Sedova et al. as the only whole-organ EP simulation study of diabetes. Possibly this was written before publication of this, but would Strocchi et al.'s conference paper "Multi-scale Whole-Heart Electromechanics Modeling to Link Cellular, Tissue and Systemic Properties to Cardiac Biomarkers in the Diabetic Male and Female Heart" be relevant?

• The review covers EP and electromechanical models, but diabetes also affects the circulatory system and hemodynamics. Are there any blood flow or circulatory models (e.g., 0D or 4D) that have been or could be used to study diabetes?

◦ Possibly relevant: Tunedal et al., "Haemodynamic effects of hypertension and type 2 diabetes: Insights from a 4D flow MRI-based personalized cardiovascular mathematical model."

• Are there any sex-specific differences of importance? Could computational models be used to capture these?

• The tables are informative and summarize key findings well. However, they can be a little overwhelming to process without knowing the field. Could they be referred to throught the discussion, when relevant? That might make the tables more integrated with the text.

• Would it be feasible to summarize findings from the tables in a schematic figure? For example, showing how ion channel changes lead to alterations in action potential, calcium transients, and active tension. Even a conceptual figure could be effective in communicating the main ideas.

• Similarly, could the impact of different drugs be visualized schematically?

Minor:

• "T2DM" is referred to in the abstract once but otherwise "Type 2 diabetes" is used; either or is fine but consistency is better.

• Should it either be "T2D" defined for "Type 2 diabetes" or "T2DM" for "Type 2 diabetes mellitus"? Again, I think either is fine but "T2DM" for "Type 2 diabetes" seems to lack a letter?

• In the schematics, I am a little confused about if this is meant to reflect on the "current use" versus "potential use". I think it's the latter, but could the authors clarify?

◦ If it is potential, would there be room for a mechanics-related output? E.g. pv-loops, which can be related to volumetric biomarkers?

• "the heterogeneity of animal models (including age, strain, mode of T2DM induction, duration of disease, concomitant conditions)" - how does strain vary between animal models? What is strain used for in this context?

• "Clinically, characteristic autonomic effects may be reflected in the ECG, notably the QT-RR slope and QTc interval prolongation" - maybe also heart rate?

• "SGLT2i have been shown to be the only drug with significant proven" - potentially this could be reformulated as something like "the only drug known to" or "to date, the only drug found to .." to avoid overstatement.

• "Largely based on Hodgkin-Huxley equations and Markov models, detailed mathematical representation" - importantly also physical laws, e.g. describing electrical propagation and mechanical stress-strain relations

• "An early computational model of T2DM was developed by Ashrafi et al." - does this refer to an ionic/cellular-level model?

• "using a novel model, T-World, that incorporates [...]" - rather than stating novelity, can the author describe what is new about it? What makes it different from the one developed by Ashrafi et al.?

• "accurately replicating the intricate branching patters of fibres" - Purkinje fibers? (Fibers would also be used for general myocytes in terms of "fiber direction", for collagen fibers, etc.)

• "These fibre networks can be optimised to fit individual patient anatomies" - I don't quite follow; is the optimisation

performed to minimize some error estimating it to be the closest fit for a given patient?

• "Wang et al. were able to recapitulate key mechanical consequences of myocardial stiffness, including changes in diastolic and systolic function, by simulating the behaviour of the ventricles in 3D using high-performance software (Santiago et al., 2018)." - the first author referred to and the reference don't match.

• "as animal models often focus on one independent factor at a time." - one can also, on the flip side, argue that animal models cannot only focus one independent factor at a time, since there will be feedback mechanisms compensating for any change; while this is something computational models can do if desired; maybe consider taking this perspective into account

• "the known or hypothesised mechanistic action of a certain compound is modelled at ionic level" - cell-level mechanics models are usually considered intracellular rather than ionic; prehaps add an "or intracellular" or "or subcellular"?

• A list of abbreviations might be helpful for the reader

END OF COMMENTS

DEPARTMENT OF COMPUTER SCIENCE
UNIVERSITY OF OXFORD

Professor Blanca Rodriguez, FESC, FEAMBES
Wellcome Trust Senior Research Fellow in Basic Biomedical Sciences
Professor of Computational Medicine
Head, Computational Biology and Health Informatics Theme
Email: blanca.rodriguez@cs.ox.ac.uk
www.cs.ox.ac.uk/ccs/people

Oxford, Thursday 6 November 2025

Dear Reviewing Editor and Reviewers of The Journal of Physiology,

We thank you for your time and commitment in reviewing our submission "Cardiac remodelling in type 2 diabetes: pathophysiological mechanisms and opportunities for computational multi-scale modelling and simulation". We are very grateful for the detailed comments and questions that you have communicated, providing constructive feedback which we believe has helped us considerably improve our manuscript.

We have provided below detailed responses to each of the reviewers' questions, and included in our responses an indication of the changes that have been implemented in the manuscript to address corresponding feedback points. We have also included four new visuals in the manuscript which we hope will increase the clarity and impact of the work. We hope that you will find these amendments satisfactory and consider our revised submission suitable for publication in The Journal of Physiology.

Once again thank you for your consideration,
With best wishes,

Ambre Bertrand, Jakub Tomek and Blanca Rodriguez

DEPARTMENT OF COMPUTER SCIENCE
UNIVERSITY OF OXFORD

Professor Blanca Rodriguez, FESC, FEAMBES
Wellcome Trust Senior Research Fellow in Basic Biomedical Sciences
Professor of Computational Medicine
Head, Computational Biology and Health Informatics Theme
Email: blanca.rodriguez@cs.ox.ac.uk
www.cs.ox.ac.uk/ccs/people

Editor

Reviewer 1 has rightly noted that the introduction does not clearly specify whether therapies are glucose-, HFpEF-, or arrhythmia-directed, and I support this request for clarification. Both reviewers emphasise the need for a unifying framework that links cellular, structural, and 3D modelling to pharmacological applications; I agree this would substantially strengthen the paper. As the reviewers have highlighted, the "in silico" section must be more balanced, with limitations acknowledged and supported by appropriate citations, including recent electromechanics and haemodynamic work. Both reviewers have stressed the lack of figures, and I strongly support adding visualisations, as well as integrating the tables more directly with the text. Finally, I support Reviewer 2's detailed technical suggestions (fibrosis terminology, LV volume changes, calcium handling, HRV, sex differences, and consistency issues), which will improve precision and readability.

REFEREE COMMENTS

Referee #1:

The manuscript provides a review of the pathophysiological mechanisms underlying electrophysiological, structural, and autonomic remodeling in type 2 diabetes (T2DM), as well as the clinical implications of these changes.

The introduction is effective in highlighting the high prevalence of T2DM and its association with increased cardiovascular risk. The authors correctly emphasize that the limited mechanistic understanding of sudden cardiac death (SCD) in T2DM poses a barrier to the development of novel therapies. However, I recommend the authors provide greater clarity regarding the therapies being referenced in this section. For instance, are these therapies aimed at glucose control, heart failure with preserved ejection fraction (HFpEF) management, or arrhythmia-specific interventions? Current HFpEF management already has well-established clinical protocols-how would the proposed novel therapies differ from or improve upon these existing approaches?

Thank you for your thorough review and for this comment. We clarified the therapeutic focus and that the main/earliest benefit that simulation-based approaches are likely to bring is aimed at reducing arrhythmic risk. Although there is strong potential for studying mechanical function

DEPARTMENT OF COMPUTER SCIENCE
UNIVERSITY OF OXFORD

Professor Blanca Rodriguez, FESC, FEAMBES
Wellcome Trust Senior Research Fellow in Basic Biomedical Sciences
Professor of Computational Medicine
Head, Computational Biology and Health Informatics Theme
Email: blanca.rodriguez@cs.ox.ac.uk
www.cs.ox.ac.uk/ccs/people

too, this is not the primary focus of this review, but we do reference this in the introduction (penultimate paragraph) in the context of multi-scale modelling.

In addition, the statement "Early identification of cardiac abnormalities in T2DM is therefore crucial to mitigate risk of heart failure and arrhythmias. In the ECG, QT segment and T wave irregularities, notably QT prolongation and T wave flattening, are common in T2DM even without overt heart failure or ischemia" gives the impression that the manuscript may also be reviewing diagnostic approaches. However, this theme is not further developed in the subsequent sections. Explicitly clarifying how these points connect to the main scope of the review would demonstrate greater expertise and strengthen the translational impact of the paper.

Thank you; we have now clarified the paragraph to avoid giving the impression that we will focus on diagnostics.

The review's strength lies in its detailed examination of both structural and subcellular electrophysiological remodeling, with an in-depth explanation of ion channel changes related to T2DM. This section is thorough and well-referenced. The manuscript would benefit from a more explicit link between these mechanistic insights and how computational modeling could bridge current knowledge gaps, for example, by identifying new therapeutic targets or stratifying patient subgroups at highest arrhythmic risk.

We now explicitly describe this point in the section “Bridging scales: human-based multi-scale computational modelling and simulation of the diabetic heart” by providing a more comprehensive introductory paragraph to the section.

The section "In silico evaluation of drug safety and efficacy" reads somewhat overconfident and lacks sufficient supporting evidence. Firstly, the authors argue that computational heart models are superior to animal models due to interspecies differences with humans. While this is an important point, the statement should be more balanced. The validation of computational heart models-particularly in the context of pharmacological development-still relies heavily on animal studies. The key advantage of computational models is that they can accelerate the exploratory phase of pharmacological discovery and are more cost-effective compared to animal experiments. Acknowledging both the strengths and the current limitations would improve the credibility of this section. Secondly, this paragraph has the potential to be one of the highlights of the review, as it directly addresses the rationale for developing a T2DM-specific cardiac model.

DEPARTMENT OF COMPUTER SCIENCE
UNIVERSITY OF OXFORD

Professor Blanca Rodriguez, FESC, FEAMBES
Wellcome Trust Senior Research Fellow in Basic Biomedical Sciences
Professor of Computational Medicine
Head, Computational Biology and Health Informatics Theme
Email: blanca.rodriguez@cs.ox.ac.uk
www.cs.ox.ac.uk/ccs/people

However, the literature cited does not sufficiently connect the concept of 3D anatomical modeling with pharmacological evaluation. If a fully developed 3D heart model that incorporates T2DM-specific cellular EP properties is not yet available, the authors could strengthen the discussion by citing analogous studies in other disease contexts where cellular remodeling has been incorporated into pharmacological modeling (e.g., PMID: 39973098). This would demonstrate that the approach is feasible and underscore its potential applicability in T2DM. Overall, I recommend that the authors expand and reframe this section to more clearly emphasize how T2DM-specific computational models could contribute to drug safety and efficacy evaluation, while providing a balanced discussion of both opportunities and current challenges.

Thank you for raising these important points. We have expanded the section to more explicitly discuss limitations as well as strengths (see introductory paragraph and explicit “Limitations” subsection in the “Bridging Scales: …” section), and emphasize the synergy with experimental studies. That said, we note that, ideally, validation of model suitability for pharmacological studies relies on comparison to human data, e.g. through the CredibleMeds database (PMID PMC5601077, PMC6970534).

We have also addressed your comment regarding the lack of clarity on the connection between multi-scale modelling and pharmacological evaluation. We now provide a more explicit update on the current state of 3D T2DM cardiac modelling in the “Modelling and simulating electrophysiological and electromechanical behaviour across scales ” subsection by referencing our recent conference work proposing the first multi-scale biophysically-detailed modelling and simulation framework for T2DM cardiac EP. We have also revised the subsection “In silico evaluation of drug safety and efficacy” : we emphasise that further development and validation are required, refer to previously presented T2DM multi-scale models, include a reformulated bridging sentence highlighting analogous studies in other disease contexts, and included the suggested reference (PMID: 39973098).

Finally, the manuscript would be strengthened by the inclusion of more visualizations. Figures illustrating structural substrates, cellular models, and examples of computational simulations would make the review more accessible and engaging for readers, especially those less familiar with the technical aspects. At present, the text is heavily descriptive, and complementary visual aids would significantly improve clarity and impact.

Thank you for the suggestion which was echoed by the other reviewer - we have now added several new visuals throughout to illustrate cellular effects, structural effects, impact of the medications discussed, and a focus explaining the computational modelling framework in more detail.

DEPARTMENT OF COMPUTER SCIENCE
UNIVERSITY OF OXFORD

Professor Blanca Rodriguez, FESC, FEAMBES
Wellcome Trust Senior Research Fellow in Basic Biomedical Sciences
Professor of Computational Medicine
Head, Computational Biology and Health Informatics Theme
Email: blanca.rodriguez@cs.ox.ac.uk
www.cs.ox.ac.uk/ccs/people

Referee #2:

Bertrand, Tomek, and Rodriguez present a thorough review of cardiac remodeling commonly observed in patients with type 2 diabetes. This is an important and interesting topic, given the elevated cardiovascular risks associated with diabetes. As the authors note, there are currently very few models to date focusing on these aspects, but the potential for further exploration is clear. I found the connections to interstitial fibrosis and ion channel remodeling particularly compelling.

I have some suggestions and questions - I hope they come across as constructive rather than critical.

Major:

• The emphasis on interstitial fibrosis is refreshing and, from a computational perspective, under-studied compared to replacement fibrosis. However, I was occasionally unsure about the distinctions:

◦ **In the schematics, there is an arrow from "increased interstitial fibrosis" to "LGE MRI." LGE MRI best captures replacement fibrosis, while T1 imaging is more suitable for interstitial fibrosis; would it be an idea to display a T1 image instead?**

Thank you for pointing this out, this is a pertinent point which we have now corrected in the schematic.

◦ **In the "Fibrosis" section, the authors alternate between "diffuse interstitial fibrosis" and the more general "fibrosis." Are all studies mentioned here focused on interstitial fibrosis?**

Yes indeed - this has now been clarified explicitly in the text.

◦ **Similarly, in the section on computational models of fibrosis, do the studies concern interstitial fibrosis specifically, or other forms?**

Yes, the studies mentioned in this section concern specifically diffuse fibrosis (aside from one which considers different types including diffuse). We have clarified this explicitly in the text.

DEPARTMENT OF COMPUTER SCIENCE
UNIVERSITY OF OXFORD

Professor Blanca Rodriguez, FESC, FEAMBES
Wellcome Trust Senior Research Fellow in Basic Biomedical Sciences
Professor of Computational Medicine
Head, Computational Biology and Health Informatics Theme
Email: blanca.rodriguez@cs.ox.ac.uk
www.cs.ox.ac.uk/ccs/people

Regarding diffuse/interstitial fibrosis: in our context, these can usually be used interchangeably. Typically, diffuse fibrosis is interstitial - the word diffuse is used here to emphasise the heterogenous appearance/distribution of fibroblasts scattered throughout the tissue, as opposed to dense/compact clumps of (replacement/focal) fibrotic tissue.

◦ **Are all computational models discussed EP studies, or do some include mechanics? What do the authors think should differ in the representation of fibrosis in EP versus mechanics in future electromechanical 3D models?**

Thank you for this interesting question. Initially the references cited here did concern fibrosis modelling in the context of computational EP studies only, but we have now added an additional short paragraph to highlight the relevance of fibrosis modelling in electromechanics studies. (see “Modelling cardiac fibrosis” section)

• **In the schematics, LV diastolic and systolic volumes are marked as "decreasing," while LV concentric remodeling is shown as "increasing." The text focuses on concentric remodeling. Could the authors describe the decreasing volume aspects?**

Thanks for the comment. Concentric remodelling refers to an increase in LV relative wall thickness with a reduction in chamber size. As LV wall thickness increases, the LV ED and ES volumes typically decrease - reflecting a geometric adaptation rather than dilatation. We have included this clarification in the text in the subsection “Anatomical remodelling” and added a visual to summarise these changes in the relevant section.

◦ **Does thickening combined with decreasing volume suggest inward thickening, where the blood pool decreases while the volume contained by the pericardium remains roughly the same?**

Yes, the observed decrease in LV volumes alongside increased wall thickness reflects inward (concentric) remodelling, reducing blood pool size while overall/external cardiac dimensions remain mostly unchanged.

◦ **Is there literature relevant for comparing systolic/diastolic volume changes under pressure (giving end-diastolic and end-systolic volumes) versus pure geometrical changes (without blood flow)?**

We have looked into this and found a paper (PMID: 37670768) that presents a unified physical model integrating both pressure-volume relations and their geometric bases. This model can be used to analyse cardiac function both under physiological pressures and based solely on geometry and tissue properties, which directly addresses comparisons between loaded and

Professor Blanca Rodriguez, FESC, FEAMBES
Wellcome Trust Senior Research Fellow in Basic Biomedical Sciences
Professor of Computational Medicine
Head, Computational Biology and Health Informatics Theme
Email: blanca.rodriguez@cs.ox.ac.uk
www.cs.ox.ac.uk/ccs/people

pure geometric changes. We have added a reference to this study in the “Modelling cardiac anatomy in 3D” subsection later on in the text - while slightly tangential we welcome the comment and believe this could be a relevant addition in context.

◦ **Does a constant LV mass index imply an increase in both LV mass and BMI, or no change in either?**

LV mass index = LV mass / body size metric (metric is usually body surface area BSA, or BMI)

Thus if LV mass index is constant, it is impossible to know whether this implies an increase in both LV mass and BMI unless we know the value of one of those two metrics.

• Although not the main focus of the review, is there any anatomical remodeling of the left atrium worth mentioning? For example, thickening of the atrial walls could be relevant in the context of atrial fibrillation.

Thank you for raising this point. While we acknowledge the relevance of atrial remodelling and its potential implications notably in AF and HFpEF in T2DM patients, it is beyond the scope of the review. As SCD arises primarily from ventricular arrhythmias, we have chosen to focus on the ventricles only.

• The variance in increased vs. decreased L-type calcium channel (ICaL) conductance is interesting, as these changes would have opposite effects on both arrhythmogenicity and contractility. Do the authors have any intuition about which trend is more prevalent? Are these changes mostly affecting maximum amplitude or baseline (diastolic) levels?

We agree that the disagreement between studies on what happens to ICaL in T2DM is interesting, given the central role of the current in excitation-contraction coupling and arrhythmia. The discrepancy likely arises from differences in disease stage, degree of development of heart failure, etc., and given the heterogeneity of patients and remodelling involved, it is very hard to determine which factor will prevail in general.

We note though, that the range of remodelling in T2DM is broad, and there are additional factors that can promote e.g. arrhythmia due to EADs. This is supported by recent results from a computational study of cellular-level mechanisms of T2DM using the new ventricular cardiomyocyte model T-World, which suggest that overall, all the T2DM remodelling scenarios tested (some with +ICaL, others with –ICaL) remain at greater risk of arrhythmia, indicating that the interplay of other ionic currents other than ICaL likely plays a role in arrhythmogenesis in T2DM (preprint PMID 40196542).

DEPARTMENT OF COMPUTER SCIENCE
UNIVERSITY OF OXFORD

Professor Blanca Rodriguez, FESC, FEAMBES
Wellcome Trust Senior Research Fellow in Basic Biomedical Sciences
Professor of Computational Medicine
Head, Computational Biology and Health Informatics Theme
Email: blanca.rodriguez@cs.ox.ac.uk
www.cs.ox.ac.uk/ccs/people

Those changes would likely have a greater effect on peak, rather than diastolic calcium levels (which may be more strongly affected by remodelling of NCX or late Na current for example).

To reflect these points in the text, we have refined the “Subcellular electrophysiological remodelling and calcium handling” subsection and have also included further discussion points n the subsection “Divergent ICaL remodelling” (previously called “PI3K/AKT signalling and ICaL”).

• While the review focuses on ECG changes, arrhythmogenicity, and diastolic dysfunction, many of the mechanisms discussed could also contribute to systolic dysfunction. This is briefly mentioned in the context of fibrosis, but other mechanisms would also impair contractility, especially those affecting intracellular calcium. Could the authors expand on this aspect?

We have addressed these points, as mentioned in more detail above in the previous question/response, and in the manuscript text in the section on cellular-level changes (referring to the effects of intracellular calcium handling on contractility more generally as opposed to diastolic dysfunction specifically), as well as the subsection heading change to “Diastolic and systolic function” in the structural remodelling section that refers to organ-level systolic dysfunction.

• I found the section about the damages to the electrical conductive system (SA/AV node and Purkinje fibers) interesting.

◦ "In the early stages of T2DM-induced cardiac autonomic neuropathy, parasympathetic function decreases relative to sympathetic function which increases, causing heart rate variability to decrease." What are the (negative) consequences of decreased heart rate variability?

Thanks for your positive comment. Decreased heart rate variability is associated with a heightened risk of adverse cardiac events (ref PMID: 8941112). We have included this sentence in the relevant paragraph.

◦ Is heart rate variability measured beat-to-beat in the same patient, or across different activities?

Heart rate variability is typically measured using ECG to identify sequential heartbeats of a patient across a given time window and analyse the variation in RR interval. This is standard clinical practice so we have opted not to provide this definition explicitly in the text.

DEPARTMENT OF COMPUTER SCIENCE
UNIVERSITY OF OXFORD

Professor Blanca Rodriguez, FESC, FEAMBES
Wellcome Trust Senior Research Fellow in Basic Biomedical Sciences
Professor of Computational Medicine
Head, Computational Biology and Health Informatics Theme
Email: blanca.rodriguez@cs.ox.ac.uk
www.cs.ox.ac.uk/ccs/people

◦ **What do the authors think about the relative impact of conduction system damage versus interstitial fibrosis on (1) arrhythmogenicity and (2) systolic function? Which do they consider more consequential?**

Thank you for a thoughtful prompt - we have reinforced explicitly the effects of fibrosis/collagen deposition on contractility and systolic function in the “Cardiac fibrosis” and “Diastolic and systolic dysfunction” subsections to address (2).

Regarding (1), we think the important difference lies in the conduction system and fibrosis’ roles as arrhythmia trigger and substrate, respectively. Conduction system damage is likely to have a more direct effect by causing slowed or heterogeneous impulse propagation, promoting conduction block and re-entry which may initiate an arrhythmia (structurally), while fibrosis will amplify this by increasing tissue-level electrical heterogeneity and conduction dispersion due to impaired electrical coupling between adjacent myocytes. We’ve included this discussion point in the “Cardiac autonomic dysfunction” subsection and hope it provides a helpful nuance of those two factors.

• **The review mentions Sedova et al. as the only whole-organ EP simulation study of diabetes. Possibly this was written before publication of this, but would Strocchi et al.'s conference paper "Multi-scale Whole-Heart Electromechanics Modeling to Link Cellular, Tissue and Systemic Properties to Cardiac Biomarkers in the Diabetic Male and Female Heart" be relevant?**

Thanks for bringing this paper to our attention (we did notice it but too late to include in our original submission…!). We have now added a reference to it in the section on multi-scale models of T2DM, in the paragraph relating to electromechanics.

• **The review covers EP and electromechanical models, but diabetes also affects the circulatory system and hemodynamics. Are there any blood flow or circulatory models (e.g., 0D or 4D) that have been or could be used to study diabetes?**

◦ **Possibly relevant: Tunedal et al., "Haemodynamic effects of hypertension and type 2 diabetes: Insights from a 4D flow MRI-based personalized cardiovascular mathematical model."**

While effects of T2DM on the wider circulatory system is beyond the scope of our article, we appreciate that the paper suggested here does provide an interesting and different perspective on the use of modelling and simulation for cardiovascular disease in T2DM. We have included a mention to this at the end of the subsection “Modelling and simulating electrophysiological and electromechanical behaviour across scales” which provides a nice avenue for future expansions of the frameworks presented.

DEPARTMENT OF COMPUTER SCIENCE
UNIVERSITY OF OXFORD

Professor Blanca Rodriguez, FESC, FEAMBES
Wellcome Trust Senior Research Fellow in Basic Biomedical Sciences
Professor of Computational Medicine
Head, Computational Biology and Health Informatics Theme
Email: blanca.rodriguez@cs.ox.ac.uk
www.cs.ox.ac.uk/ccs/people

• Are there any sex-specific differences of importance? Could computational models be used to capture these?

Yes to both - we had omitted this aspect altogether in our original submission for the sake of brevity but considering its importance and your comment we have now added a new subsection in the "Bridging Scales" section, highlighting key differences in structural/cellular remodelling in M vs F in T2DM, very briefly mentioning their clinical implications, and highlighting some examples of modelling studies where sex differences have been incorporated and how this could be applied to extend the T2DM modelling frameworks.

• The tables are informative and summarize key findings well. However, they can be a little overwhelming to process without knowing the field. Could they be referred to throught the discussion, when relevant? That might make the tables more integrated with the text.

Thanks for raising this, we now point the reader toward individual tables at various relevant points throughout the text.

• Would it be feasible to summarize findings from the tables in a schematic figure? For example, showing how ion channel changes lead to alterations in action potential, calcium transients, and active tension. Even a conceptual figure could be effective in communicating the main ideas.

We have included a summary of key changes in Figure 2 and hope that the detailed text in the relevant subsection (including new additions and clarifications arising from the reviews) clarify the complex links between those changes, complexities in calcium handling, and subsequent biomarker changes observed.

• Similarly, could the impact of different drugs be visualized schematically?

Yes, we have also included a visualisation recapitulating the impact of the drugs discussed, see new Figure 3.

Minor:

• "T2DM" is referred to in the abstract once but otherwise "Type 2 diabetes" is used; either or is fine but consistency is better.

Professor Blanca Rodriguez, FESC, FEAMBES
Wellcome Trust Senior Research Fellow in Basic Biomedical Sciences
Professor of Computational Medicine
Head, Computational Biology and Health Informatics Theme
Email: blanca.rodriguez@cs.ox.ac.uk
www.cs.ox.ac.uk/ccs/people

We have changed “T2DM” to “type 2 diabetes” for simplicity and to avoid non-essential acronyms in the abstract.

• Should it either be "T2D" defined for "Type 2 diabetes" or "T2DM" for "Type 2 diabetes mellitus"? Again, I think either is fine but "T2DM" for "Type 2 diabetes" seems to lack a letter?

We have chosen throughout the main text to abbreviate type 2 diabetes mellitus as T2DM.

• In the schematics, I am a little confused about if this is meant to reflect on the "current use" versus "potential use". I think it's the latter, but could the authors clarify?

Yes, the graphical abstract figure mainly highlights the scope of potential use cases of T2DM cardiac modelling for T2DM, with a focus on electrophysiology, although we have expanded this slightly to account for your suggestion below.

◦ If it is potential, would there be room for a mechanics-related output? E.g. pv-loops, which can be related to volumetric biomarkers?

See response above plus changes made to updated graphical abstract and new Figure 4.

• "the heterogeneity of animal models (including age, strain, mode of T2DM induction, duration of disease, concomitant conditions)" - how does strain vary between animal models? What is strain used for in this context?

For example, the induction of diabetes with streptozotocin and subsequent disease progression differs in mice of different strain e.g. ICR mouse vs. ddY mouse. We have added an explicit reference in the text to a study that highlights this (see PMID 16755002).

• "Clinically, characteristic autonomic effects may be reflected in the ECG, notably the QT-RR slope and QTc interval prolongation" - maybe also heart rate?

Correct - while we already refer to the effects of autonomic dysfunction on heart rate/heart rate variability earlier in this subsection, we have reminded this explicitly in the sentence you have highlighted for completeness.

• "SGLT2i have been shown to be the only drug with significant proven" - potentially this could be reformulated as something like "the only drug known to" or "to date, the only drug found to .." to avoid overstatement.

Professor Blanca Rodriguez, FESC, FEAMBES
Wellcome Trust Senior Research Fellow in Basic Biomedical Sciences
Professor of Computational Medicine
Head, Computational Biology and Health Informatics Theme
Email: blanca.rodriguez@cs.ox.ac.uk
www.cs.ox.ac.uk/ccs/people

Thank you for the suggestion - we have revised this sentence which now reads “To date, SGLT2i are the only medication type found to have significant proven benefit in patients with HFpEF.”

• "Largely based on Hodgkin-Huxley equations and Markov models, detailed mathematical representation" - importantly also physical laws, e.g. describing electrical propagation and mechanical stress-strain relations

We agree that these factors are important when it comes to the modelling of the whole heart, but in this particular section, we discuss models underlying ionic channels at the cellular level.

• "An early computational model of T2DM was developed by Ashrafi et al." - does this refer to an ionic/cellular-level model?

Correct, we have now made this explicit in the relevant sentence.

• "using a novel model, T-World, that incorporates [...]" - rather than stating novelity, can the author describe what is new about it? What makes it different from the one developed by Ashrafi et al.?

We now clarified this section and emphasized the difference in baseline cellular model and the remodelling incorporated.

• "accurately replicating the intricate branching patters of fibres" - Purkinje fibers? (Fibers would also be used for general myocytes in terms of "fiber direction", for collagen fibers, etc.)

Thanks for pointing this out - indeed, the two studies referred to here focus on modelling Purkinje fibres. We have made this explicit in the relevant sentence.

• "These fibre networks can be optimised to fit individual patient anatomies" - I don't quite follow; is the optimisation performed to minimize some error estimating it to be the closest fit for a given patient?

Thank you for raising this ambiguity, we have revised the sentence to make the message clearer. It now reads: “These models can be further personalised using optimisation-based methods to generate patient-specific Purkinje fibre networks that best fit an individual’s given cardiac anatomy and reproduce realistic activation timings.”

• "Wang et al. were able to recapitulate key mechanical consequences of myocardial stiffness, including changes in diastolic and systolic function, by simulating the

DEPARTMENT OF COMPUTER SCIENCE
UNIVERSITY OF OXFORD

Professor Blanca Rodriguez, FESC, FEAMBES
Wellcome Trust Senior Research Fellow in Basic Biomedical Sciences
Professor of Computational Medicine
Head, Computational Biology and Health Informatics Theme
Email: blanca.rodriguez@cs.ox.ac.uk
www.cs.ox.ac.uk/ccs/people

behaviour of the ventricles in 3D using high-performance software (Santiago et al., 2018)." - the first author referred to and the reference don't match.

Apologies for the confusion; the reference of Santiago et al. referred to the high performance software mentioned at the end of this sentence. We have split the sentence into two parts with relevant references at the end of each respective one to avoid the confusion. It now reads:

“Wang et al. were able to recapitulate key mechanical consequences of myocardial stiffness, including changes in diastolic and systolic function, by simulating the behaviour of the ventricles in 3D (Wang et al., 2021). Simulations were achieved using the high-performance software, Alya, developed for coupled multi-physics and multi-scale problems (Santiago et al., 2018).”

• "as animal models often focus on one independent factor at a time." - one can also, on the flip side, argue that animal models cannot only focus one independent factor at a time, since there will be feedback mechanisms compensating for any change; while this is something computational models can do if desired; maybe consider taking this perspective into account

Thank you for your pertinent perspective - we have amended the relevant section “In silico evaluation of drug safety and efficacy” to account for this.

• "the known or hypothesised mechanistic action of a certain compound is modelled at ionic level" - cell-level mechanics models are usually considered intracellular rather than ionic; prehaps add an "or intracellular" or "or subcellular"?

We have changed “ionic” to “intracellular”.

• A list of abbreviations might be helpful for the reader

A helpful suggestion which we have taken on board. A list of key abbreviations can now be found following the abstract.

Dear Ms Bertrand,

Re: JP-TR-2025-287140R1 "Cardiac remodelling in type 2 diabetes: pathophysiological mechanisms and opportunities for multi-scale computational modelling and simulation" by Ambre Bertrand, Jakub Tomek, and Blanca Rodriguez

We are pleased to tell you that your paper has been accepted for publication in The Journal of Physiology.

TRANSPARENT PEER REVIEW POLICY: To improve the transparency of its peer review process, The Journal of Physiology publishes online as supporting information the peer review history of all articles accepted for publication. Readers will have access to decision letters, including Editors' comments and referee reports, for each version of the manuscript, as well as any author responses to peer review comments. Referees can decide whether or not they wish to be named on the peer review history document.

The last Word (or similar) version of the manuscript provided will be used by the Production Editor to prepare your proof. When this is ready you will receive an email containing a link to Wiley's Online Proofing System. The proof should be thoroughly checked and corrected as promptly as possible.

Authors should note that it is too late at this point to offer corrections prior to proofing. Major corrections at proof stage, such as changes to figures, will be referred to the Editors for approval before they can be incorporated. Only minor changes, such as to style and consistency, should be made at proof stage. Changes that need to be made after proof stage will usually require a formal correction notice.

All queries at proof stage should be sent to: TJP@wiley.com.

Are you on Twitter? Once your paper is online, why not share your achievement with your followers? Please tag The Journal (@jphysiol) in any tweets and we will share your accepted paper with our 30,000 followers!

Yours sincerely,

Laura Bennet
Senior Editor
The Journal of Physiology

P.S. - You can help your research get the attention it deserves! Check out Wiley's free Promotion Guide for best-practice recommendations for promoting your work at www.wileyauthors.com/eeo/guide. You can learn more about Wiley Editing Services which offers professional video, design, and writing services to create shareable video abstracts, infographics, conference posters, lay summaries, and research news stories for your research at www.wileyauthors.com/eeo/promotion.

• IMPORTANT NOTICE ABOUT OPEN ACCESS: To assist authors whose funding agencies mandate immediate public access to published research findings, The Journal of Physiology allows authors to pay an Open Access (OA) fee to have their papers made freely available immediately on publication.

The Corresponding Author will receive an email from Wiley with details on how to register or log-in to Wiley Authors Services where you will be able to place an order.

You can check if your funder or institution has a Wiley Open Access Account here: https://authorservices.wiley.com/author-resources/Journal-Authors/licensing-and-open-access/open-access/author-compliance-tool.html.

EDITOR COMMENTS

Reviewing Editor:

Thank you for submitting the revised version of your manuscript and for your thorough responses to the reviewers' comments.

REFEREE COMMENTS

Referee #1:

No more comments.

Referee #2:

Thank you for thoroughly addressing all points, and especially I appreciate the added figures, which enhances the main points of the review!
